# Non Uniqueness of Power-Law Flows

**DOI:** 10.1007/s00220-021-04231-7

**Published:** 2021-10-06

**Authors:** Jan Burczak, Stefano Modena, László Székelyhidi

**Affiliations:** 1 Institut für Mathematik, Universität Leipzig, 04103 Leipzig, Germany; 2 Fachbereich Mathematik, Technische Universität Darmstadt, 64285 Darmstadt, Germany

## Abstract

We apply the technique of convex integration to obtain non-uniqueness and existence results for power-law fluids, in dimension $d\ge 3$. For the power index *q* below the compactness threshold, i.e. $q \in (1, \frac{2d}{d+2})$, we show ill-posedness of Leray–Hopf solutions. For a wider class of indices $q \in (1, \frac{3d+2}{d+2})$ we show ill-posedness of distributional (non-Leray–Hopf) solutions, extending the seminal paper of Buckmaster & Vicol [10]. In this wider class we also construct non-unique solutions for every datum in $L^2$.

## Introduction

This paper studies non-uniqueness and existence of solutions of the following model of non-Newtonian flows in *d* dimensions, $d \ge 3$

1 $$\begin{aligned} \begin{aligned} \partial _{t}v + \mathrm{div} \,(v\otimes v) - \mathrm{div} \,\mathcal {A} (Dv )+\nabla \tilde{\pi }&= 0, \\ \mathrm{div} \,v&= 0,&\\ v_{t =0}&= v_0, \end{aligned} \end{aligned}$$

where the velocity field *v* and and the pressure $\tilde{\pi }$ are the unknowns, $Dv = \frac{1}{2} (\nabla v + \nabla ^T v)$, and the non-Newtonian tensor $\mathcal {A}$ is given by the following power law

2 $$\begin{aligned} \mathcal {A} (Q) = (\nu _0+\nu _1 |Q|)^{q-2} Q, \end{aligned}$$

for some $\nu _0, \nu _1 \ge 0$ and $q \in (1, \infty )$. A natural energy associated with the system (1) is

3 $$\begin{aligned} e(t) = \int |v(t)|^2 + 2 \int _0^t \int \mathcal {A} \big (D v (s)\big ) D v (s) ds. \end{aligned}$$

Let us consider a distributional solution *v* to (1), (2) with spatial mean zero, on a *d*-dimensional flat torus. The formula (3) together with $\mathcal {A} (Q) Q \sim |Q|^q$ explains why $v \in L^\infty (L^2) \cap L^q (W^{1,q})$ is called an *energy solution*. If such solution satisfies additionally the energy inequality $e(t) \le e(0)$ (*t*-a.e.), then it is called a *Leray–Hopf solution*.

For the problem (1) we show two non-uniqueness and one existence result. In short:

(A) In the regime $1< q<2d/(d+2)$: There are non-unique Leray–Hopf solutions.
(B) In the regime $1< q < (3d+2)/(d+2)$: There are non-unique distributional solutions dissipating the kinetic part of the energy.
(C) In the regime $1< q < (3d+2)/(d+2)$: For any initial datum $a \in L^2$ there are infinitely many distributional solutions of the Cauchy problem.

Our results are sharp concerning the power-law index *q*. The regime $1< q < (3d+2)/(d+2)$ includes the case of the incompressible Navier–Stokes equation in $d\ge 3$. The precise formulations can be found in Sect. 1.3.

### Background of power-law flows

Model (1) with a slightly different choice of $\mathcal {A}(Q)$, namely

4 $$\begin{aligned} \mathcal {A}(Q) = (\nu _0+\nu _1 |Q|^{q-2}) Q, \end{aligned}$$

with $q\ge 2$ was introduced to wide mathematical community by Ladyzhenskaya at her 1966 Moscow ICM speech; her formula (30) in [24] corresponds exactly to (1), (4). With $q=2$, both models (1), (2) and (1), (4) reduce to the (incompressible) Navier–Stokes equations.

The Ladyzhenskaya’s choice: (4) with $q \ge 2$ and our (2) with $q \ge 2$ are analytically equivalent. In particular, the non-Newtonian tensor $\mathcal {A}(Q)$ is in both cases nonsingular at $Q=0$, and distributional solutions are well-defined for velocity fields in the class

5 $$\begin{aligned} v \in L^2_{loc}, \quad Dv \in L^q_{loc}. \end{aligned}$$

The difference between (4) and (2) plays a role for $q < 2$. Firstly, $\nu _0+\nu _1 |Q|^{q-2}$ of (4) is singular at $|Q|=0$, while our $(\nu _0+\nu _1 |Q|)^{q-2}$ for $\nu _0>0$ is not. More importantly, in (4) a *linear* dissipation is present. Thus, distributional solutions to (1)–(4) make sense provided $Dv \in L^2_{loc}$. So the choice (2) isolates the ‘pure $L^q$-dissipation’ behaviour, while (4) involves ‘$L^2$-$L^q$ dissipation’.

Ladyzhenskaya’s rationale for analysing (1) was twofold: on the one hand, relaxation $q \ge 2$ helps to avoid the traps of the Navier–Stokes case $q =2$. At the same time, the choice of power-laws for the tensor $\mathcal {A}$ is both consistent with first principles of continuum mechanics and widely used in applications. Let us elaborate on each of these points.

The model (1) with power-law for $\mathcal {A}$ of type (2) or (4) agrees with the constitutive relations for incompressible, viscous fluids. Recall that in deriving the Navier–Stokes equation one restricts the admissible relations between the Cauchy stress tensor $\mathcal {T}$ and *D* (dictated by the material frame indifference) by the Ansatz of linear dependence between $\mathcal {T}$ and *D* (i.e. by the Stokes law), cf. [22]. The power law model relaxes this Ansatz, but remains well within the frame indifference principle.

Of course studying an arbitrary model that is merely consistent with the first principles may be applicationally void. This is not the case of (1) however. The power-laws have been proposed independently in 1920’s by Norton [33] in metallurgy and by de Waele [19] and Ostwald [34] in polymer chemistry. The related timeline can be found in section 1 of [36]. For details, the interested reader may consult also the monographs [3, 28, 37, 38] and the recent survey [4] with its references. Just in order to fix the hydrodynamical intuition, let us observe that $q<2$ in (1) models the case when the fluid is more viscous (roughly, ‘solid-like’) for small shears (‘external forces’) and less viscous (‘liquid-like’) for large shears e.g. ice pack, ketchup, emulsion paints, hair gel, whereas $q>2$ means reverse behavior e.g. cornstarch-water solution, silicone-based solutions.

Let us note that, despite the mathematical interest in $q \ge 2$ in context of gaining regularity compared to Navier–Stokes equations, the ‘shear-thinning’ case $q \le 2$ appears to be more meaningful for applications, where models of type (1) with $\nu _0>0$ appear as Bird-Carreau-Yasuda models (or called by a subset of those names). In particular, experimental fits for the threshold value 6/5 and above can be found on p. 174 of [3]. Furthermore, even parameter choices well-into our Leray–Hopf non-uniqueness regime are suggested, cf. p.18 of [39]. (In both [3] and [39] $n=q-1$, $d=3$. A discrepancy between appearing there *a* and our model is insignificant for our results.)

From the applicational perspective, our result may be seen as invalidating certain choices of parameters and data.

### Essential analytical results for power-law fluids

Consider the system (1), (2). For $q > \frac{2d}{d+2}$ the space $W^{1,q}$ of system’s energy embeds compactly into $L^2_{loc}$ of the convective term $\mathrm{div} \,(v\otimes v)$. Hence one may expect an existence proof of Leray–Hopf solutions via compactness methods. Indeed, a relevant statement can be found in [20], which is itself the final step in a chain of attempts of many authors, including Frehse and Nečas with collaborators [21, 29] to improve the lower bound on *q*. To be precise, the energy inequality $e(t) \le e(0)$ is not stated explicitly in [20]; however it can be proven e.g. along the lines of proof of Theorem 3.3 of [4].

Observe that (1) with $\nu _0 =0$ is invariant under the scaling

6 $$\begin{aligned} v_\lambda := \lambda ^\alpha v ( \lambda x, \lambda ^{\alpha +1} t) \quad \text { with } \; \alpha = \frac{q-1}{3-q}. \end{aligned}$$

Consequently, the energy of $v_\lambda$ vanishes on small scales iff $q < \frac{3d+2}{d+2}$. This suggests that the case $q \ge \frac{3d+2}{d+2}$ of (1) is a perturbation of the problem (1) without the convective term. Indeed, for $q \ge \frac{3d+2}{d+2}$ uniqueness in the energy class (at least for tame initial data) holds, cf. [28], section 5.4.1; see also [11].

What is known about existence and uniqueness of solutions to (1) can be thus sketched as follows

**Fig. 1:**
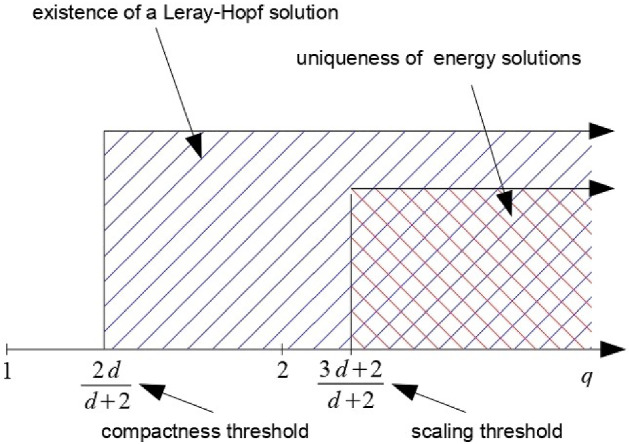
Known results

### Our contribution

The short version of our results presented at the very beginning of the paper, recast graphically to facilitate comparison with Fig. 1, reads

**Fig. 2:**
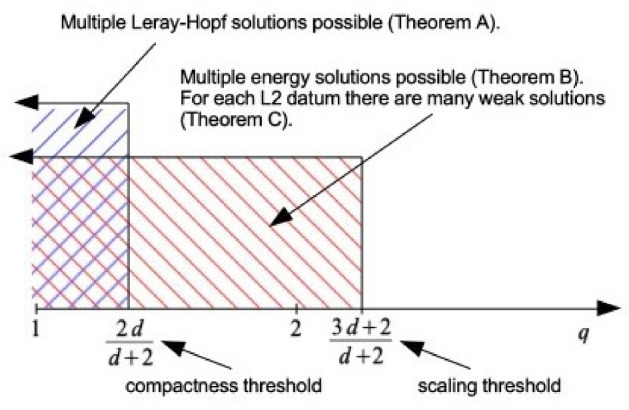
Our results

Observe that Fig. 2 complements Fig. 1 sharply with respect to *q*.

Let us now present the detailed statements of our results. We always consider system (1) on the *d*-dimensional flat torus $\mathbb {T}^d$, with *v* having its spatial mean zero, and we define the notion of distributional solution in the usual way.

#### Definition 1

Let $q \in (1,\infty )$. A vector field $v \in L^2_{loc}(\mathbb {T}^d \times (0,1) )$ with $Dv \in L_{loc}^{\max \{1,q-1\}} (\mathbb {T}^d \times (0,1))$ is a *distributional solution* to (1), (2) on the space-time domain ${\mathbb {T}^{d}}\times (0,1)$ if

$$\begin{aligned} \int _0^1 \int _{\mathbb {T}^{d}}-v \cdot \partial _{t}\varphi - v\otimes v \nabla \varphi + \mathcal {A} (Dv ) \nabla \varphi = 0, \qquad \forall _{t \in [0,1]} \quad \int _{\mathbb {T}^{d}}v (t) \cdot \nabla \psi = 0 \end{aligned}$$

for any divergence-free $\varphi \in C^1 ({\mathbb {T}^{d}}\times [0,1])$ vanishing at $t=0$ and $t=1$, and any $\psi \in C^1 ({\mathbb {T}^{d}})$.

The condition $Dv \in L_{loc}^{\max \{1,q-1\}}$ guarantees that $\mathcal {A}(Dv) \in L^1_{loc}$.

#### Non-uniqueness in the Leray–Hopf class

Our first theorem and its corollary show that below the compactness exponent, i.e. for $q<\frac{2d}{d+2}$, multiple Leray–Hopf solutions may emanate from the same $L^2$ initial data. In fact, we produce solutions $v \in C(L^2) \cap C (W^{1,q})$ with quite arbitrary pre-determined profile *e* of the (total) energy (3).

##### Theorem A

Consider (1), (2) on the space-time domain ${\mathbb {T}^{d}}\times (0,1)$. Let $q< \frac{2d}{d+2}$. Fix an arbitrary $e \in C^\infty ([0,1]; [1/2,1])$. There exists $v \in C([0,1]; L^2(\mathbb {T}^d)) \cap C ([0,1]; W^{1,q}(\mathbb {T}^d))$ such that

1. *v* solves (1) distributionally;
2. the total energy equals *e*, i.e.
  7 $$\begin{aligned} \int _{\mathbb {T}^{d}}|v|^2 (t) + 2 \int _0^t \int _{\mathbb {T}^{d}}\mathcal {A} (D v) D v = e(t). \end{aligned}$$

Moreover, fix $$0 \le T_1 < T \le 1$$ and two energy profiles $$e_1, e_2$$ as above, such that $$e_1 (t)= e_2 (t) $$ for $$t \in [0, T]$$. There exists $$v_1, v_2$$ satisfying (1), (2) and such that $$v_1 (t)= v_2 (t)$$ for $$ t \in [0, T_1]$$. In particular, choosing $$T_1 = 0$$, $$T = 1/2$$ and $$e_1, e_2$$ to be as above, non-increasing and $$e_1 \not \equiv e_2$$, the corresponding $$v_1, v_2$$ are two distinct Leray–Hopf solutions with the same initial datum.

Analysing the proof of Theorem A one realises that choosing an infinite family of non-increasing energy profiles $\{e_\alpha \}_{\alpha \in A}$ with a common $C^1$ bound, one can produce infinitely many distinct Leray–Hopf solutions with the same initial datum.

#### Non-uniqueness of distributional solutions

If we drop the ambition to control the energy and require only to pre-determine the profile of the kinetic part of the energy $\int _{\mathbb {T}^{d}}|v|^2 (t)$, then we produce non-unique solutions for exponents below the scaling-critical one, i.e. for $q<\frac{3d+2}{d+2}$. Moreover, they enjoy the regularity $v \in C(L^2) \cap C (W^{1,r})$ for any $r<\frac{2d}{d+2}$. This is our second result.

##### Theorem B

Consider (1), (2) on ${\mathbb {T}^{d}}\times (0,1)$. Let $q< \frac{3d+2}{d+2}$. Fix any $e \in C^\infty ([0,1]; [1/2,1])$ and $r \in (\max \{1, q-1\}, \frac{2d}{d+2})$. There exists null-mean $v \in C([0,1]; L^2(\mathbb {T}^d)) \cap C ([0,1]; W^{1,r}(\mathbb {T}^d))$ such that

1. *v* solves (1) distributionally;
2. the kinetic energy equals *e*, i.e.
  8 $$\begin{aligned} \int _{\mathbb {T}^{d}}|v|^2 (t) = e(t). \end{aligned}$$

Moreover, fix $$0 \le T_1 < T \le 1$$ and two energy profiles $$e_1, e_2$$ as above, such that $$e_1 (t)= e_2 (t) $$ for $$t \in [0, T]$$. There exists $$v_1, v_2$$ satisfying 1), 2) and such that $$v_1 (t)= v_2 (t)$$ for $$ t \in [0, T_1]$$. In particular, choosing $$T_1 = 0, T =1/2$$ and $$e_1, e_2$$ to be as above, non-increasing and $$e_1 \not \equiv e_2$$, the corresponding $$v_1, v_2$$ are two distinct distributional solutions, which belong to $$C (L^2) \cap C (W^{1,r})$$, dissipate the kinetic energy, and share the same initial datum.

#### Existence of multiple solutions for any $L^2$ data

In Theorems A, B the initial data are attained strongly (in particular we can add initial values to the distributional formulas for solutions, extending test functions to non-vanishing ones at $t=0$), but they are constructed in the convex integration scheme, thus possibly non-generic. This issue is addressed in our third theorem. It shows existence of energy solution emanating from *any* solenoidal vector field in $L^2$, for power laws below the scaling exponent.

##### Theorem C

Consider (1), (2) on ${\mathbb {T}^{d}}\times (0,1)$. Let $q< \frac{3d+2}{d+2}$, $r \in (\max \{1, q-1\}, \frac{2d}{d+2})$. Fix an arbitrary nonzero $v_0 \in L^2(\mathbb {T}^d)$, $\mathrm{div} \,v_0 = 0$. There exist continuum of $v \in C((0,1]; L^2(\mathbb {T}^d)) \cap L^r ((0,1); W^{1,r}(\mathbb {T}^d))$ such that

1. *v* solves (1) distributionally;
2. $v|_{t=0} = v_0$, in the sense that as $t \rightarrow 0^+$, $v(t) \rightarrow v_0$ weakly in $L^2$ and strongly in $L^{q_0}$, any $q_0 <2$.

##### Remark 1

Theorems A, B, C hold for Ladyzhenskaya’s choice (4) instead of our choice (2), in the case $q \ge 2$. On the contrary, for $q<2$, they do not hold for (4), since (4) contains also *linear* dissipation (see Sect. 1.1). However, Ladyzhenskaya’s interest was limited to $q \ge 2$ (as a vehicle to mitigate Navier–Stokes difficulties), and in this range the theorems hold both for (2) and for (4), whereas, for $q < 2$, our choice (2) is more common in applications than (4).

### Differences between our non-uniqueness and existence results

The non-uniqueness Theorems A, B, focus on possibly strongest notions of solutions: they allow, respectively, for full- or kinetic energy inequality and strong attainment of a (constructed) initial datum, but they do not produce non-unique solutions for any initial datum. Conversely, Theorem C provides existence of many weak solutions for an arbitrary solenoidal initial datum in $L^2$. In particular, this is the first existence proof for the case of $q \le \frac{2d}{d+2}$. The obtained solutions are, however, much weaker than that of Theorems A, B : they do not allow for any kind of energy inequality (in fact, even their kinetic energies are in a sense pathologically large) and the initial datum is attained merely in a weak sense.

For the Euler equations a result similar to our Theorem C (existence of distributional solutions for *any* initial datum, with unnaturally high energy) was first proven in [40]. The difference with our Theorem C is that, in the case of the Euler equations (no dissipation), the problem of controlling higher order derivatives is completely absent.

### The 3d Navier–Stokes case

Theorems B, C cover also the case of non-unique weak solutions of three-dimensional Navier–Stokes equations, first proven in [10]. Our Theorem B shows that $\nabla v \in L^{6/5-}$. This probably holds for solutions constructed in [10] as well, though the best regularity claimed there is $\mathrm{curl \,}v \in L^1$. Theorem C produces infinitely many weak solutions for any divergence-free datum in $L^2$ (but with unnaturally high energies).

### Methodology and plan

Our approach follows the convex integration methods introduced to inviscid fluid dynamics in [18, 25], culminating in [7, 23], and extended to the Navier–Stokes case in the important paper [10]. Results on a system involving fractional laplacian can be found in [16, 26, 35]. Other related interesting results include [2, 8, 9, 12–15].

We stay close to the concentration-oscillation method developed for the transport equation in [31, 32], and localised to avoid dimension loss in [30], see also [5].

The basic picture of the construction, as in any convex integration scheme applied to the equations of fluid dynamics, is the following. Given an exact flow $(v, \pi )$, i.e. a solution to (1), one tries to distinguish the good (‘laminar’, ‘averaged’) component of *v*, i.e. $\langle v \rangle$ and the remainder, thought to be responsible for turbulence (interestingly, the case $q=3$ in (2), where scaling (6) fails, is the Smagorinsky model for turbulence). A typical averaging process $\langle \cdot \rangle$ does not commute with nonlinear quantities, thus applying $\langle \cdot \rangle$ to (1) yields for $u = \langle v\rangle$

$$\begin{aligned}&\partial _{t}u + \mathrm{div} \,(u\otimes u) - \mathrm{div} \,\mathcal {A} (Du ) +\nabla \langle \pi \rangle \\&\quad = \mathrm{div} \,\big (u\otimes u - \langle v \otimes v \rangle \big ) - \mathrm{div} \,\big ( \mathcal {A} (Du ) - \langle \mathcal {A} (Dv ) \rangle \big ) =: \mathrm{div} \,R. \end{aligned}$$

Above, *u* is a well-behaved flow and the Reynolds stress *R* encodes the difference between $u = \langle v\rangle$ and the exact *v* itself. The rough idea behind producing non-unique solutions to (1) is to reverse-engineer the above picture. We can thus consider the following relaxation of (1)

9 $$\begin{aligned} \begin{aligned} \partial _{t}u + \mathrm{div} \,(u\otimes u) - \mathrm{div} \,\mathcal {A} (Du ) +\nabla \pi&= -\mathrm{div} \,R, \\ \mathrm{div} \,u&= 0. \end{aligned} \end{aligned}$$

Assume we have identity (9) with certain $(u_0,\pi _0,R_0)$. It is easy to find at least one smooth solution of (10), since $R_0$ is at our disposal. If one can produce another $u_1, q_1$ such that $(u_1,q_1,R_1)$ solves (9) and $R_1$ is strictly smaller than $R_0$, there is a hope to iteratively diminish the Reynolds part $R_n$ to 0 with $n \rightarrow \infty$. Consequently, in the limit one produces an exact solution $v,\pi$. Non uniqueness in the above procedure may be specified in at least two ways:

- either by enforcing *v* to be equal at some times, say for $t \in [0,1/3]$, to a given regular solution $v_1$ and for $t \in [2/3,1]$ to another regular solution $v_2$, as for instance in [6] or [31].
- or by specifying a kinetic energy profile, see e.g. [10, 18], or the present work.

### Organisation of proofs

In Sect. 2, we state the main proposition of the paper, i.e. Proposition 1, which contains the inductive step described above, from $(u_0, \pi _0, R_0)$ to $(u_1, \pi _1, R_1)$, with $R_1$ “much smaller” than $R_0$. Section 3 gathers preliminary material. In Sect. 4 we introduce a generalisation of Mikado flows that serves as a building block for $u_1$ given $u_0$. Next, in Sect. 5, assuming a solution $(u_0, \pi _0, R_0)$ to (9) is given, we define $(u_1, \pi _1, R_1)$. Estimates for $(u_1-u_0)$ and $R_1$ occupy Sect. 6. Section 7 concludes the proof of the main Proposition 1. Having it in hand, we prove Theorem A in Sect. 8. The proofs of Theorems B–C follow similar lines and therefore are only sketched in Sects. 9–10.

### Notation

We use mostly standard notation, e.g. ${\mathbb {T}^{d}}$ denotes the *d*-dimensional torus $[0, 1]^d$, $\dot{W}^{1,q}$ is a homogenous Sobolev space, $C_0^\infty (\mathbb {T}^d; B)$ are smooth functions with mean zero, domain $\mathbb {T}^d$ and values in set *B* (the target set will be sometimes omitted). We take $\mathbb {N}=\{1,2, \dots \}$.

We suppress the variables and the spatial domain of integration, if no confusion arises. We use $| \cdot |_.$ instead of $\Vert \cdot \Vert _.$ for norms. For $L^p$-norms on the torus ${\mathbb {T}^{d}}$, we will abbreviate $|\cdot |_{L^p({\mathbb {T}^{d}})}$ to $| \cdot |_{L^p}$ or even to $| \cdot |_{p}$. In other cases, e.g. when taking the $L^p$-norm on ${\mathbb {R}}^d$, we will explicitly write the underlying domain, where the norm is calculated, e.g. $|\cdot |_{L^p({\mathbb {R}}^d)}$. The finite-dimensional norm is $| \cdot |$. The projection onto null-mean functions is $P_{\ne 0}f:= f - \fint _{\mathbb {T}^{d}}f$.

We will call $d \times d$ (symmetric) matrices *(symmetric) tensor*. For a tensor *T*, we denote its traceless part by $\mathring{T} := T - \frac{1}{d} \mathrm{tr \,}(T) {\mathrm{Id}}$. The space of symmetric tensors will be denoted by $\mathcal {S}$, its open subset of positive definite tensors by $\mathcal {S}_+$. If *R* is a symmetric tensor, $\mathrm{div} \,R$ is the usual row-wise divergence.

We use two types of constants *M*’s, which are uniform over iterations, and *C*’s which are not (both possibly with subscripts), for details see Sect. 6.1. All constants may vary between lines.

Further notation is introduced locally when needed.

## Main Proposition: An Iteration Step

Recall that $\mathcal {S}$ is the space of symmetric tensors.

### Definition 2

A solution to *the Non-Newtonian-Reynolds system* is a triple $(u,\pi ,R)$ where

$$\begin{aligned} u \in C^\infty ([0,1] \times \mathbb {T}^d; \mathbb {R}^d), \quad \pi \in C([0,1] \times \mathbb {T}^d; \mathbb {R}) \quad R \in C([0,1] \times \mathbb {T}^d; \mathcal S) \end{aligned}$$

with spatial null-mean $u,\pi$, satisfying

10 $$\begin{aligned} \begin{aligned} \partial _{t}u + \mathrm{div} \,(u\otimes u) - \mathrm{div} \,\mathcal {A} (Du ) +\nabla \pi&= -\mathrm{div} \,\mathring{R}, \\ \mathrm{div} \,u&= 0. \end{aligned} \end{aligned}$$

in the sense of distributions.

### Remark 2

Despite smoothness of *u*, we can not require that (10) is satisfied in the classical sense or $\pi ,R$ are smooth (in space), because of non-smoothness of $\mathcal {A} (Du)$.

### Remark 3

(*R* vs $\mathring{R}$) Use of the trace-free Reynolds stress simplifies computation, in particular proof of the energy iterate Proposition 14. The difference between *R* to $\mathring{R}$ is facilitated by the ambiguity of pressure: $(u,\pi ,R)$ solves (10) $\iff (u,\pi -\frac{1}{d} tr R,\mathring{R})$ solves (10).

As observed in the introduction, the crucial point in the convex integration scheme is, given $(u_0, q_0, R_0)$, to produce an appropriate correction $(u_1,q_1,R_1)$ which decreases $R_i$, improves the energy gap, and retains as much regularity as possible. This single iteration step is given by

### Proposition 1

Let $\nu _0, \nu _1 \ge 0$ and $q< \frac{2d}{d+2}$ be fixed. Fix an arbitrary $e \in C^\infty ([0,1]; [\frac{1}{2},1])$. There exist a constant *M* such that the following holds.

Let $(u_0, \pi _0, R_0)$ be a solution to the Non-Newtonian-Reynolds system (10), as in Definition 2. Let us choose any $\delta , \eta , \epsilon \in (0,1]$. Assume that

11 $$\begin{aligned} \frac{3}{4} \delta e(t) \le e(t) - \left( \int _{\mathbb {T}^{d}}|u_0|^2 (t) + 2 \int _0^t \int _{\mathbb {T}^{d}}\mathcal {A} (D u_0) D u_0 \right) \le \frac{5}{4} \delta e(t) \end{aligned}$$

and

12 $$\begin{aligned} |\mathring{R}_0 (t)|_{L^1} \le \frac{\delta }{2^7 d}. \end{aligned}$$

Then, there is another solution $(u_1, \pi _1, R_1)$ to (10) (as in Definition 2) such that

13a $$\begin{aligned}&|(u_1 - u_0)(t)|_{L^2} \le M \delta ^\frac{1}{2} \end{aligned}$$

13b $$\begin{aligned}&|(u_1 - u_0)(t) |_{W^{1,q}} \le \eta \end{aligned}$$

13c $$\begin{aligned}&|R_1 (t)|_{L^1} \le \eta . \end{aligned}$$

Furthermore

14 $$\begin{aligned} \frac{3}{8} \delta e(t) \le e(t) - \left( \int _{\mathbb {T}^{d}}|u_1|^2 (t) + 2 \int _0^t \int _{\mathbb {T}^{d}}\mathcal {A} (D u_1) D u_1 \right) \le \frac{5}{8} \delta e(t). \end{aligned}$$

## Preliminaries

### Control of $\mathcal {A}$

We collect the needed growth estimates for $\mathcal {A} (Q)$ and for $\mathcal {A} (Q)Q$.

#### Lemma 1

(Growth estimates for $\mathcal {A}$). Let $\mathcal {A} := (\nu _0 + \nu _1 |Q|)^{q-2}Q$, with $\nu _0, \nu _1 \ge 0$. Then

15 $$\begin{aligned}&|\mathcal {A} (Q) - \mathcal {A} (P)| \le \left\{ \begin{aligned}&C_{\nu _1} |Q-P|^{q-1}&\text { for } \nu _0=0, q \le 2 \\&C_{\nu _0} |Q-P|&\text { for } \nu _0>0, q \le 2 \\&C_{q, \nu _0, \nu _1} |Q-P| \left( 1 + |Q|^{q-2} + |P|^{q-2} \right)&\text { for } q\ge 2 \end{aligned} \right. \end{aligned}$$

16 $$\begin{aligned}&|\mathcal {A} (Q)Q - \mathcal {A} (P)P| \le C_{q, \nu _0, \nu _1} \big (1 + |Q|^{q-1} + |P|^{q-1} \big ) |Q-P|. \end{aligned}$$

The proof is standard. For convenience of the reader, we added it in Appendix.

#### Remark 4

Lemma 1 extends to other tensors $\mathcal {A}$, e.g. $(\nu _0+\nu _1 |Q|^2)^\frac{q-2}{2} Q$, or to ones given by an appropriate *N*-function. Consequently, our result extends to such tensors.

### Nash-type decomposition

Let us denote the set of positive-definite $d \times d$ symmetric tensors by ${\mathcal {S}} _+$. We recall Lemma 2.4 in [17]

#### Lemma 2

For any compact set ${\mathcal {N}} \subset {\mathcal {S}} _+$ there exists a finite set $K \subset {\mathbb {Z}^{d}}$ and smooth functions $\Gamma _k: {\mathcal {N}} \rightarrow [0,1]$, such that any $R \in {\mathcal {N}}$ has the following representation:

$$\begin{aligned} R = \sum _{k \in K} \Gamma ^2_k (R) k \otimes k. \end{aligned}$$

### The role of oscillations

The convex integration paradigm is to use fast oscillations of corrector functions (correcting $u_i$ to $u_{i+1}$ in our case, roughly speaking) to inductively diminish error terms (in our case Reynolds stresses $R_i$). Thus for a function *f* and $\lambda \in \mathbb {N}$ let us define

$$\begin{aligned} f_\lambda (x) := f (\lambda x). \end{aligned}$$

Observe that $f_\lambda$ has the same $L^p$ norms as *f* since we work on ${\mathbb {T}^{d}}$, and a factor $\lambda$ appears for each derivative, i.e.

$$\begin{aligned} |\nabla ^s f_\lambda |_p = \lambda ^s | \nabla ^s f|_p, \quad s \in \mathbb {N}\cup \{0\}. \end{aligned}$$

It holds

#### Proposition 2

(Mean value). Let $a \in C^\infty (\mathbb {T}^d; \mathbb {R})$, $v \in C_0^\infty (\mathbb {T}^d; \mathbb {R})$. Then for any $r \in [1, \infty ]$

17 $$\begin{aligned} \Big |\int _{\mathbb {T}^{d}}a v_\lambda \Big | \le \lambda ^{-1} C_r |\nabla a|_{r} |v|_{r'} \end{aligned}$$

#### Proof

The case $r=\infty$ follows the proof of Lemma 2.6 in [31]. For the case $r<\infty$, since *v* is null-mean, let us solve the Laplace equation $\mathrm{div} \,\nabla h = v$ and define $G:= \nabla h$. It holds $(\mathrm{div} \,G)_\lambda = \lambda ^{-1} \mathrm{div} \,(G_\lambda )$ and thus, integrating by parts and using Hölder

$$\begin{aligned} \Big |\int _{\mathbb {T}^{d}}a v_\lambda \Big | = \lambda ^{-1} \Big |\int _{\mathbb {T}^{d}}a \mathrm{div} \,G_\lambda \Big | \le \lambda ^{-1} |\nabla a|_r |G_\lambda |_{r'} = \lambda ^{-1} |\nabla a|_r |G|_{r'} \end{aligned}$$

The Sobolev embedding for the null-mean *G* yields $|G|_{r'} \le C |\nabla G|_{L^{\min (r',d+1)}} = C|\nabla ^2 h|_{L^{\min (r',d+1)}}$. This is controlled thanks to Calderón-Zygmund theory by $|v|_{L^{\min (r',d+1)}}$.

$$\square $$

Even when the l.h.s. of (17) is replaced with $\int _{\mathbb {T}^{d}}|a v_\lambda |$, the decorrelation between frequencies of *a* and $v_\lambda$ allows to improve the generic Hölder inequality to (for the proof cf. Lemma 2.1 of [31]):

#### Proposition 3

(Improved Hölder). Let *f*, *g* be smooth maps on $\mathbb {T}^d$. Let $r \in [1,\infty ]$. Then

18 $$\begin{aligned} |f g_\lambda |_r \le |f|_r |g|_r + C_r \lambda ^{-\frac{1}{r}} |f|_{C^1} |g|_r. \end{aligned}$$

### Antidivergence operators

We provide now various inverse divergence operators, needed for construction of $R_1$ in Proposition 1, with appropriate estimates. The purpose of the bilinear inverse divergences below is to extract oscillations of one function, say $g_\lambda$, out of the product $fg_\lambda$. The last of them, ${\mathcal {R}}^2_N$, is an operator with symmetric tensor values, such that $\mathrm{div} \,\mathrm{div} \,{\mathcal {R}}^2_N f = f$ for every null-mean real function *f*; it facilitates construction of the $R_{lin}$ term of $R_1$, cf. (60).

#### Proposition 4

Let $$p,r,s \in [1,\infty ]$$ and $$\frac{1}{p} = \frac{1}{s} + \frac{1}{r}$$.

(i) ($\mathrm {div}^{-1}$: symmetric antidivergence) There exists $\mathrm {div}^{-1}: C_0^\infty (\mathbb {T}^d; \mathbb {R}^d) \rightarrow C_0^\infty (\mathbb {T}^d; {\mathcal {S}} )$ such that $\mathrm{div} \,\mathrm {div}^{-1}u = u$ and for $i\ge 0$ one has
  19 $$\begin{aligned} | \nabla ^i \mathrm {div}^{-1}u|_{p} \le C_{k,p} |\nabla ^i u|_{{p}} , \end{aligned}$$
  and for the fast oscillating $u_\lambda$
  20 $$\begin{aligned} | \nabla ^i \mathrm {div}^{-1}u_\lambda |_{p} \le C_{k,p} \lambda ^{i-1} |\nabla ^i u|_{p} . \end{aligned}$$
(ii) (${\mathcal {R}}_N$: improved symmetric bilinear antidivergence) For any $N \ge 1$ there exists a bilinear operator ${\mathcal {R}}_N: C^\infty (\mathbb {T}^d; \mathbb {R}) \times C^\infty _0(\mathbb {T}^d; \mathbb {R}^d) \rightarrow C^\infty _0(\mathbb {T}^d; {\mathcal {S}} )$ such that $\mathrm{div} \,{\mathcal {R}}_N (f, u) = f u - \fint fu$ and
  21 $$\begin{aligned} | \mathcal {R}_N (f, u_\lambda ) |_{p} \le C_{d,p,s,r,N} | u |_{s} \left( \frac{1}{\lambda } |f|_{r} + \frac{1}{\lambda ^N} |\nabla ^N f|_{r} \right) . \end{aligned}$$
(iii) ($\tilde{\mathcal {R}}_N$: improved symmetric bilinear antidivergence on tensors) For any $N \ge 1$ there exists a bilinear operator $\tilde{\mathcal {R}}_N: C^\infty (\mathbb {T}^d; \mathbb {R}^d) \times C^\infty _0(\mathbb {T}^d; \mathbb {R}^{d \times d}) \rightarrow C^\infty _0(\mathbb {T}^d; {\mathcal {S}} )$ such that $\mathrm{div} \,\tilde{\mathcal {R}}_N (v,T) = Tv - \fint Tv$ and
  22 $$\begin{aligned} | \tilde{\mathcal {R}}_N (v, T_\lambda ) |_{p} \le C_{d,p,s,r,N} |T|_{s} \left( \frac{1}{\lambda } |v|_{r} + \frac{1}{\lambda ^N} |\nabla ^N v|_{r} \right) . \end{aligned}$$
(iv) (${\mathcal {R}}^2_N$: improved symmetric bilinear double antidivergence) For any $N \ge 1$ there exists a bilinear operator ${\mathcal {R}}^2_N: C^\infty (\mathbb {T}^d; \mathbb {R}) \times C^\infty _0(\mathbb {T}^d; \mathbb {R}) \rightarrow C^\infty _0(\mathbb {T}^d; {\mathcal {S}} )$ such that $\mathrm{div} \,\mathrm{div} \,\mathcal {R}^2_N (f,g) = fg - \fint fg$ and for any $j \in \mathbb {N}\cup \{0\}$
  23 $$\begin{aligned} |\nabla ^{j} {\mathcal {R}}^2_N (f, g_\lambda )|_p \le C_{j,d,p,s,r,N} \lambda ^j |g|_{W^{j,s}} \left( \frac{1}{\lambda ^{2}} |f|_{r} + \frac{1}{\lambda ^{N}} |\nabla ^N f|_{r} + \frac{1}{\lambda ^{2N+j}} |\nabla ^{2N +j} f|_{r} \right) . \end{aligned}$$

The proof is standard, cf. [18, 30, 31] and can be found in Appendix.

#### Remark 5

(${\mathcal {R}}^2_N \ne {\mathcal {R}}_N \circ {\mathcal {R}}_N$) For the operator defined in (iv), we use the notation ${\mathcal {R}}^2_N$ to denote that this operator acts as a double antidivergence. It does not coincide in general with ${\mathcal {R}}_N \circ {\mathcal {R}}_N$.

#### Remark 6

(${\mathcal {R}}_\infty$) The above bilinear antidivergences may be thought of as approximations of ‘ideal antidivergence’ operators $\mathcal {R}_\infty$, $\mathcal {R}^2_\infty$ satisfying

24 $$\begin{aligned} | \mathcal {R}_\infty (f, u_\lambda ) |_{p} \lesssim \frac{1}{\lambda } | u |_{s} |f|_r, \qquad | \nabla ^j \mathcal {R}^2_\infty (f, g_\lambda ) |_{p} \lesssim \lambda ^{j-2} |g |_{W^{j,s}} |f|_r, \end{aligned}$$

where the gap between ${\mathcal {R}}_N$ and ${\mathcal {R}}_\infty$ closes as $N \rightarrow \infty$, similarly for ${\mathcal {R}}^2_N$ and ${\mathcal {R}}^2_\infty$.

## Mikado Flows

In this section we introduce the building blocks of our construction, namely the *concentrated localized traveling Mikado flows*. This section is essentially a rearrangement of known material: *Mikado flows* were first introduced in [17] in the framework of the Euler equations; the *concentrated Mikado flows* (or *fields*) were defined in [31], inspired by the *intermittent Beltrami waves* of [10]; the *traveling* version of concentrated Mikados we present here is close in spirit to the *intermittent jets* introduced for the first time in [6], and resemble the construction in [30].

The original Mikado flows of [17] are fast oscillating pressureless stationary solutions to Euler equations having the form

25 $$\begin{aligned} \Psi _\lambda ^k(x) k = \Psi ^k(\lambda x) k, \end{aligned}$$

where $k \in {\mathbb {Z}^{d}}$ is a direction. For a finite set of directions *K* (given by the decomposition Lemma 2) one can choose functions $\Psi ^k \in C^\infty _0({\mathbb {T}^{d}}, \mathbb {R})$ so that the following holds for any $k, k' \in K$ and $\lambda \in \mathbb {N}$

26 $$\begin{aligned} (i)\qquad&\mathrm{div} \,\Psi _\lambda ^k k= 0, \nonumber \\ (ii)\qquad&\mathrm{div} \,( \Psi _\lambda ^k k \otimes \Psi _\lambda ^k k) = 0, \nonumber \\ (iii)\qquad&\fint _{\mathbb {T}^{d}}(\Psi ^k)^2 = 1 \quad \text { thus } \quad \fint _{\mathbb {T}^{d}}\Psi _\lambda ^k k \otimes \Psi _\lambda ^k k = k \otimes k, \nonumber \\ (iv)\qquad&\Psi _\lambda ^k k \quad \text { and } \quad \Psi _\lambda ^{k'} k' \quad \text { have disjoint supports for }k \ne k'. \end{aligned}$$

Satisfying property (i) is equivalent to choosing $\Psi ^k$ so that $\nabla \Psi ^k \cdot k \equiv 0$, then also (ii) follows. Having (iii) is a normalisation of $\fint _{\mathbb {T}^{d}}(\Psi ^k)^2$. Disjointness of supports (iv) is ensured in $d\ge 3$ via an appropriate choice of an anchor point $\zeta _k$ for the cylinder $B_\rho (0) + \{ \zeta _k + s k \}_{s \in \mathbb {R}} + {\mathbb {Z}^{d}}$ (which is the periodisation of the cylinder $B_\rho (\zeta _k) + \{ s k\}_{s \in \mathbb {R}}$ with radius $\rho$ and axis being the line passing through $\zeta _k$ with direction *k*). Such choice is possible in view of the first part of the following lemma.

### Lemma 3

(Disjoint periodic tubes and blobs) There exist $\{\zeta _k\}_{k \in K} \subseteq \mathbb {R}^d$ and $\rho >0$ such that for all $k,k' \in K$, $k \ne k'$:

(i) if $$d \ge 3$$,
  27 $$\begin{aligned} \big ( B_\rho (\zeta _k) + s k + {\mathbb {Z}^{d}}\big ) \cap \big ( B_\rho (\zeta _{k'}) + s' k' + {\mathbb {Z}^{d}}\big ) = \emptyset \end{aligned}$$
  for every $$s,s' \in \mathbb {R}$$;
(ii) if $$d = 2$$,
  28 $$\begin{aligned} \big ( B_\rho (\zeta _k) + s k + \mathbb {Z}^2 \Big ) \cap \big ( B_\rho (\zeta _{k'}) + sk' + \mathbb {Z}^2 \Big ) = \emptyset \end{aligned}$$
  for every $$s \in \mathbb {R}$$.

Notice the difference between (27) and (28): in (27) there are two free parameters $s,s'$, whereas in (28) there is only one free parameter *s*.

Part (ii) of the statement is not strictly needed for our proof (because we are always assuming $d \ge 3$). We think however that Part (ii) could be of interest in view of future applications to two dimensional problems, and thus we decided to include it in the statement. Proof of the Lemma can be found in Appendix.

The convex integration approach uses the properties (i)-(iv) to diminish a given Reynolds stress $R_0$ of a given solution $(u_0, \pi _0, R_0)$ to the Non-Newtonian-Reynolds system (10) by correcting $u_0$ roughly as follows. Thanks to (iii), we can decompose $R_0$ via the Nash Lemma 2 into

29 $$\begin{aligned} \sum _{k} \Gamma ^2_k (R_0) k \otimes k = \sum _{k} \Gamma ^2_k (R_0) \fint _{\mathbb {T}^{d}}\Psi _\lambda ^k k \otimes \Psi _\lambda ^k k. \end{aligned}$$

Let us add to $u_0$ the corrector $\tilde{u} = \Gamma _k (R_0) \Psi _\lambda ^k k$. Recall the notation $P_{\ne 0}f:= f - \fint _{\mathbb {T}^{d}}f$. Thanks to (iv) and (ii)

$$\begin{aligned} \mathrm{div} \,\big ( \tilde{u} \otimes \tilde{u} - R_0 \big )= \sum _{k} P_{\ne 0}\left( \Psi _\lambda ^k k \otimes \Psi _\lambda ^k k \right) \nabla \Gamma ^2_k (R_0) . \end{aligned}$$

Since the term $P_{\ne 0}\left( \Psi _\lambda ^k k \otimes \Psi _\lambda ^k k \right)$ is $\lambda$-periodic and null mean, applying $\mathcal {R}_\infty$ of (24) to the r.h.s. above yields $R_1$ of order $\lambda ^{-1}$, such that $\mathrm{div} \,R_1 = \mathrm{div} \,(\tilde{u} \otimes \tilde{u} - R_0)$. So picking $\lambda$ large, i.e. letting $\Psi _\lambda ^k$ oscillate fast, allows to deal with the error $R_0$. The property (i) allows to control $\mathrm{div} \,\tilde{u}$.

### Concentrated Mikado flows

Since

$$\begin{aligned} |\nabla ^i \Psi ^k_\lambda k|_{L^p({\mathbb {T}^{d}})} = \lambda ^i |\nabla ^i \Psi ^k k|_{L^p({\mathbb {T}^{d}})}=C \lambda ^i \rightarrow \infty \quad \text { as } \quad \lambda \rightarrow \infty , \end{aligned}$$

fast oscillations, in general, blow up derivatives of the corrector $\tilde{u}$. Thus controlling Sobolev norms of velocity fields appearing over convex integration steps seems problematic. This issue may be circumvented by a *concentration* mechanism, introduced in [31] and critically inspired by [10].

Let us briefly explain it. For $n \le d$, take a compactly supported smooth function $f: \mathbb {R}^n \rightarrow \mathbb {R}$, rescale it to $f_\mu (x) = \mu ^a f(\mu x)$, $\mu \ge 1$, and periodize without renaming to $f_\mu : \mathbb {T}^d \rightarrow \mathbb {R}$. This is *concentrating* and results in

$$\begin{aligned} |\nabla ^i f_\mu |_{L^p(\mathbb {T}^d)} = \mu ^{a+i - \frac{n}{p}} |\nabla ^i f|_{L^p(\mathbb {R}^n)} =C \mu ^{a+i - \frac{n}{p}} \quad \text { and } \quad |\nabla ^i f_{\mu ,\lambda }|_{L^p(\mathbb {T}^n)} = C\lambda ^i\mu ^{a+i - \frac{n}{p}}. \end{aligned}$$

This procedure yields the ‘concentrated Mikado’ $\Psi _\mu ^k k$ satisfying

$$\begin{aligned} |\nabla ^i \Psi _\mu ^k k |_{L^p(\mathbb {T}^d)} = C \lambda ^i \mu ^{a+i-\frac{n}{p}}. \end{aligned}$$

Having now an interplay between $\lambda$ and $\mu$ one can expect to control certain Sobolev norms by choosing *a*, *p* appropriately. However, to preserve the properties (i) and (ii) of (26), i.e. $\nabla f_\mu \cdot k \equiv 0$ (or in other words: $\Psi _\mu ^k k$ being the Euler flow), the underlying function $f_\mu$ cannot depend on the direction *k*. It means that the underlying real function is not compactly supported in $\mathbb {R}^d$, but at best in $\mathbb {R}^{d-1}$. Thus at best $n=d-1$, but then

$$\begin{aligned} |\nabla ^i \Psi _\mu ^k k |_{L^p(\mathbb {T}^d)} = C \lambda ^i \mu ^{a+i-\frac{d-1}{p}}. \end{aligned}$$

The quantity $\fint _{\mathbb {T}^{d}}\Psi _\mu ^k k \otimes \Psi _\mu ^k k$ shall be of order $k \otimes k$, cf. (29). Therefore $\fint |\Psi _\mu ^k|^2$ should be $\lambda$- and $\mu$-independent, cf. (iii) of (26). This leads to the choice $a=\frac{d-1}{2}$ above and consequently to

30 $$\begin{aligned} (v)\qquad \qquad |\nabla ^i \Psi _\mu ^k k |_{L^p(\mathbb {T}^d)} = C \lambda ^i \mu ^{\frac{d-1}{2}+i-\frac{d-1}{p}}. \end{aligned}$$

Scaling (30) would force us to prove our results with *d* substituted by $d-1$, so that e.g. *q* could vary only in the interval $1<q< \frac{2(d-1)}{(d-1)+2}$ (which in particular requires $d \ge 4$, as in [27]).

Summing up, the concentrated Mikado $\Psi _\mu ^k k$ satisfies properties (26) (i)–(iv) of the original Mikado, but has unsatisfactory scaling (v).

### Concentrated localized Mikado flows

A natural idea to deal with the ‘loss of dimension’ in (30) is to localise the Ansatz (25). Let us thus take a smooth radial cutoff function $\phi$ and define $\Phi : \mathbb {R}^d \rightarrow \mathbb {R}$ via $\Phi (x) =\phi (|x|)$. We want to retain gains stemming from concentrating, and since now $\Phi : \mathbb {R}^d \rightarrow \mathbb {R}$, while $\Psi$ of (25) allowed merely for $d-1$ concentrations, it is better to concentrate in $\Phi$, thus producing $\Phi _\mu$. We periodize this function without renaming it and allow to oscillate at an independent frequency $\lambda _1$. Hence our new Ansatz reads

31 $$\begin{aligned} \Psi _{\lambda _2}^k \Phi _{\mu , \lambda _1} k. \end{aligned}$$

Let us now state and prove a result gathering needed properties of the cutoff $\Phi _{\mu , \lambda _1}$.

#### Lemma 4

Let $K \subset {\mathbb {Z}^{d}}$ be a fixed finite set of directions. There exists $\rho >0$ such that for every $\lambda _1 \in \mathbb {N}, \mu \in \mathbb {N}, \mu \ge \rho ^{-1}$ there is $\Phi ^k_{\mu , \lambda _1} \in C^\infty ({\mathbb {T}^{d}}; \mathbb {R})$ with the following properties

32 $$\begin{aligned}&\displaystyle \fint _{{\mathbb {T}^{d}}} (\Phi ^k_{\mu , \lambda _1})^2 dx = 1, \qquad |\Phi ^k_{\mu , \lambda _1}|_{W^{i,r}({\mathbb {T}^{d}})} \le M_{i,r,k} \lambda _1^i \mu ^{i+\frac{d}{2} - \frac{d}{r}},\end{aligned}$$

33 $$\begin{aligned}&\displaystyle {\mathrm{supp}\,}\Phi ^k _{\mu , \lambda _1}(\, \cdot \, - sk) \cap {\mathrm{supp}\,}\Phi ^{k'}_{\mu , \lambda _1}(\, \cdot \, - sk') = \emptyset \quad \text { for all } \quad k, k' \in K, k \ne k', s \in \mathbb {R}.\nonumber \\ \end{aligned}$$

#### Proof

Take $\Phi \in C^\infty _c(\mathbb {R}^{d})$, with $\mathrm{supp}\, \Phi \subseteq B_1(0) \subseteq \mathbb {R}^{d}$ such that $\int _{\mathbb {R}^{d}} \Phi ^2 = 1$. Let us concentrate $\Phi$ to $\Phi _\mu : {\mathbb {T}^{d}}\rightarrow \mathbb {R}$, hence $\fint _{{\mathbb {T}^{d}}} (\Phi _{\mu })^2 = \mu ^{a - \frac{d}{2}}$. Choosing $a= \frac{d}{2}$ yields the desired $\fint _{{\mathbb {T}^{d}}} \Phi _{\mu }^2 dx = 1$ and $|\Phi _{\mu }|_{W^{i,r}({\mathbb {T}^{d}})} \le C_{i,r} \mu ^{i+\frac{d}{2} - \frac{d}{r}}$. Concentration gives also ${\mathrm{supp}\,}\Phi _{\mu } \subseteq B_{1/\mu }(0) + \mathbb {Z}^d$.

Let $\rho >0$ and $\{\zeta _k\}_{k \in K}$ be given by Lemma 3. We now set for every $k \in K$ and $\lambda _1 \in \mathbb {N}$,

$$\begin{aligned} \Phi _{\mu , \lambda _1}^k (x) := \Phi _\mu ( \lambda _1( x- \zeta _k)). \end{aligned}$$

Property (32) now follows from the scaling properties of $\Phi _\mu$, whereas (33) from ${\mathrm{supp}\,}\Phi _{\mu } \subseteq B_{1/\mu }(0) + \mathbb {Z}^d$, (27), and assumed $\mu \ge \rho ^{-1}$ . $\square$

### Concentrated localized traveling Mikado flow

Unsurprisingly, introducing *d*-dimensional cutoff $\Phi$ destroys the properties (i)–(iii) of standard Mikados. The most severe loss, due to its critical scaling, is not having (ii) anymore. A crucial idea how to handle this issue, introduced in [10], is to let the cutoff function $\Phi$ travel in time along $l^k$ with speed $\omega$. This leads to a corrector term $Y^k$ (see below), whose time derivative compensates lack of (ii). At the same time $Y^k$ is of order $\frac{1}{\omega }$, so it can be controlled by choosing $\omega$ large.

The concentrated localized traveling Mikado flow is our final Ansatz. It will be denoted by $W^k$, but it is important to bear in mind that it is determined by the parameters

$$\begin{aligned} \mu , \lambda _1, \lambda _2, \omega \in \mathbb {N}. \end{aligned}$$

The next proposition concerns our final Mikado flows $W^k$ and Mikado correctors $Y^k$.

#### Proposition 5

Let $K \subset {\mathbb {Z}^{d}}$ be a fixed finite set of directions. Let $\Psi _{\lambda _2}^k$ be the function used to produce the standard Mikado (25) with its properties (i)–(iv). Let $\Phi _{\mu , \lambda _1}$ be the localisation provided by Lemma 4.

Define the functions $W^k: {\mathbb {T}^{d}}\times [0,1] \rightarrow {\mathbb {R}}^d$, $Y^k: {\mathbb {T}^{d}}\times [0,1] \rightarrow {\mathbb {R}}^d$ by

34 $$\begin{aligned} W^k (x,t):= \left( \Psi _{\lambda _2}^k \Phi ^k_{\mu , \lambda _1} k \right) (x -\omega t k), \qquad Y^k (x,t):= \left( \frac{1}{\omega }(\Psi ^k_{\lambda _2})^2 (\Phi _{\mu , \lambda _1}^k)^2 k \right) (x - \omega t k). \end{aligned}$$

There exists $\rho >0$ such that for every $\mu , \lambda _1, \lambda _2, \omega \in \mathbb {N}$ satisfying

35 $$\begin{aligned} \mu \ge \frac{1}{\rho }, \quad \frac{\lambda _2}{\lambda _1} \in \mathbb {N}\quad \text { and } \quad \frac{\lambda _1 \mu }{\lambda _2} < \frac{1}{2} \end{aligned}$$

the functions $W^k, Y^k$ are spatially $\lambda _1$-periodic are have the following properties:

36 $$\begin{aligned}&(v') \qquad |W^k(t)|_{W^{i,r}({\mathbb {T}^{d}})} \le M_{i,r} {\lambda _2}^i \mu ^{\frac{d}{2} - \frac{d}{r}}, \qquad |Y^k(t)|_{W^{i,r}({\mathbb {T}^{d}})} \le M_{i,r} \frac{ {\lambda _2}^i \mu ^{d - \frac{d}{r}}}{\omega }; \end{aligned}$$

37 $$\begin{aligned}&(iii') \qquad \Big | \fint W^k(t) \otimes W^k(t) - k \otimes k \Big | \le M_1 \frac{\lambda _1 \mu }{{\lambda _2}}; \end{aligned}$$

38 $$\begin{aligned}&(iv') \qquad \text { for } k, k' \in K, \; k \ne k' \mathrm{supp}\, W^k \cap \mathrm{supp}\, W^{k'}= \emptyset ; \nonumber \\&(ii') \qquad \partial _t Y^k + \mathrm{div} \,(W^k \otimes W^k) = 0. \end{aligned}$$

#### Proof

The spatial $\lambda _1$-periodicity of $W^k, Y^k$ follows from the assumption ${\lambda _2}/\lambda _1 \in \mathbb {N}$. Since $W^k, Y^k$ are obtained from stationary functions by means of a Galilean shift, for (36) and (37) it suffices to estimate the respective stationary functions.

39 $$\begin{aligned} |\Psi _{\lambda _2}^k \Phi ^k_{\mu , \lambda _1} k |_{W^{i,r}(\mathbb {T}^d)}\le & {} M_k \sum _{j=0}^i |\Psi _{\lambda _2}|_{W^{i-j,\infty }({\mathbb {T}^{d}})} |\Phi _{\mu ,\lambda _1}|_{W^{j,r}({\mathbb {T}^{d}})} \nonumber \\\le & {} M_{i,r,k} \sum _{j=0}^i \lambda _1^j \mu ^{j + \frac{d}{2} - \frac{d}{r}} {\lambda _2}^{i-j} \end{aligned}$$

with the second inequality due to (25) and (32). Since by assumption $\lambda _1 \mu < {\lambda _2}$, we obtain (36) estimate for $W^k$. A similar computation yields the estimate for $Y^k$. For (37) we compute

$$\begin{aligned} \left| \fint (\Psi ^k_{\lambda _2})^2 (\Phi _{\mu , \lambda _1}^k)^2(k \otimes k) - (k \otimes k) \right| = \left| (k \otimes k) \fint (\Phi _{\mu , \lambda _1}^k)^2 \left( (\Psi ^k_{\lambda _2})^2 - 1 \right) \right| =:I \end{aligned}$$

with the equality valid because the normalisation of (32) holds. Since the normalisation (iii) of the standard Mikado $\Psi ^k$ implies that $(\Psi ^k_{\lambda _2})^2 - 1$ is null-mean, and it oscillates at the frequency $\lambda _2$, by Proposition 2 we have

$$\begin{aligned} I \le \frac{M_k}{{\lambda _2}} \left| \nabla \left( (\Phi _{\mu , \lambda _1}^k)^2 \right) \right| _{1} |(\Psi ^k)^2-1|_{\infty } \le M_k \frac{\lambda _1 \mu }{{\lambda _2}} \end{aligned}$$

via (32). We reached (37).

Disjointness of supports of follows from (33): Assume that for some $(x,t) \in {\mathbb {T}^{d}}\times [0,1]$ and $k,k' \in K$, $k \ne k'$, $W^k(x,t) W^{k'}(x,t) \ne 0$. Hence in view of the definition (34)

$$\begin{aligned} x - \omega t k \in \mathrm{supp}\, \Phi _{\mu , \lambda _1}^k, \quad x - \omega t k' \in \mathrm{supp}\, \Phi _{\mu , \lambda _1}^{k'}, \end{aligned}$$

thus contradicting (33).

The Mikado functions have the form

$$\begin{aligned} W^k(x,t) = F(x - \omega t k) k, \quad Y^k (x,t) = \frac{1}{\omega } G(x - \omega t k) k, \end{aligned}$$

with $F^2 = G$. Therefore $\mathrm{div} \,(W^k \otimes W^k) = \big ( \nabla G(x - \omega t k) \cdot k \big ) k$, whereas $\partial _t Y^k= - \big (\nabla G(x - \omega t k) \cdot k \big ) k$. Hence (38). $\square$

#### Remark 7

Let us compare our $W^k$ with the concentrated Mikado.

- $W^k$ is not divergence free, i.e. (i) does not hold. Furthermore $W^k$ is now time dependent.
- $W^k$ does not satisfy (ii). There appears Mikado corrector $Y^k$ to compensates this deficiency, see (38). This means however that the new term $Y^k$ must be appropriately estimated.
- Property (iii) holds approximately, see (37).
- Supports are pairwise disjoint (now in space-time).
- The scaling with a dimension loss (30) is now improved to (36), which is our main gain.

Proposition 5 yields the following estimates in relation to (b), (c), (e):

$$\begin{aligned} \begin{aligned}&\left| Y^k(t) \right| _{L^2({\mathbb {T}^{d}})} \sim \frac{\mu ^{d/2}}{\omega }, \quad&\left| \nabla Y^k(t) \right| _{L^q({\mathbb {T}^{d}})} \sim \lambda _2 \frac{\mu ^{d-\frac{d}{q}}}{\omega }, \\&\left| \fint W^k(t) \otimes W^k(t) - k \otimes k \right| \sim \frac{\lambda _1 \mu }{{\lambda _2}}, \quad&|\nabla W^k(t)|_{L^q({\mathbb {T}^{d}})} \sim {\lambda _2} \mu ^{\frac{d}{2} - \frac{d}{q}}. \end{aligned} \end{aligned}$$

In order to deal with (a), let us recall the heuristics of an ideal antidivergence operator ${\mathcal {R}}_\infty$ of Remark 6. A short computation involving (34), (36), and (32) yields

$$\begin{aligned} \left| {\mathcal {R}}_\infty (\mathrm{div} \,W^k) \right| _{L^2({\mathbb {T}^{d}})} \sim \frac{\lambda _1 \mu }{{\lambda _2}}, \qquad \left| {\mathcal {R}}_\infty \partial _t W^k \right| _{L^1({\mathbb {T}^{d}})} \sim \frac{\lambda _1 \mu }{{\lambda _2}} \cdot \frac{\omega }{\mu ^{d/2}} \end{aligned}$$

Therefore, seeking smallness of

40 $$\begin{aligned} \frac{\mu ^{d/2}}{\omega }, \quad \frac{\lambda _1 \mu }{{\lambda _2}}, \quad {\lambda _2} \mu ^{\frac{d}{2} - \frac{d}{q}}, \quad \frac{\lambda _1 \mu }{{\lambda _2}} \cdot \frac{\omega }{\mu ^{d/2}} \end{aligned}$$

will motivate the choice of the relations between the parameters $\mu , \lambda _1, \lambda _2, \omega$ in Sect. 7.

Notice that we did not add the term $\lambda _2 \frac{\mu ^{d-\frac{d}{q}}}{\omega }$ to the list (40), as it is the product of the first and the third term in (40), and thus its smallness is implied by smallness of these terms. $\square$

## Definition of $(u_1, \pi _1, R_1)$

Let $(u_0, \pi _0, R_0)$ be a solution to the Non-Newtonian-Reynolds system (10), and $\delta , \eta \in (0,1]$ as in Proposition 1. We define

$$\begin{aligned} u_1 := u_0 + u_{p}+ u_{c}, \quad \pi _1 := \pi _0 + \pi _p, \end{aligned}$$

where

- $u_{p}$ is a perturbation based on the Mikado flows of Sect. 4, aimed at decreasing $R_0$,
- $u_{c}$ is a corrector restoring solenoidality of $u_1$ and compensating for our Mikado flows not solving Euler equations.

Since $(u_0, \pi _0, R_0)$ solves (10), it holds in the sense of distributions

41 $$\begin{aligned}&\partial _{t}u_1 + \mathrm{div} \,(u_1\otimes u_1) - \mathrm{div} \,\mathcal {A} (Du_1 )+\nabla \pi _1 \nonumber \\&\quad =\partial _{t}(u_{p}+ u_{c}) + \mathrm{div} \,(u_0 \otimes u_{p}+ u_{p}\otimes u_0) \nonumber \\&\qquad + \mathrm{div} \,(u_0 \otimes u_{c}+ u_{c}\otimes u_0 + u_{p}\otimes u_{c}+ u_{c}\otimes u_{p}+ u_{c}\otimes u_{c}) \nonumber \\&\qquad + \mathrm{div} \,(u_{p}\otimes u_{p}- \mathring{R}_0) \nonumber \\&\qquad - \mathrm{div} \,\left( \mathcal {A} \big (D (u_0 + u_{p}+ u_{c}) \big ) - \mathcal {A} (D u_{0}) \right) + \nabla \pi _p. \end{aligned}$$

### Decomposition of $R_0$ and energy control

In general, $R_0$ is only continuous (recall Definition 2 and Remark 2). Since it is convenient to work with smooth objects, we regularize $R_0$ (extended for times outside [0, 1] by $R_0(x,0)$ and $R_0(x,1)$, respectively) with the standard mollifier $\phi _\epsilon$ in space and time. Thus

42 $$\begin{aligned} \mathring{R}_0^\epsilon := \mathring{R}_0 * \phi _\epsilon . \end{aligned}$$

Now $R_0^\epsilon$ is smooth and (12) implies

43 $$\begin{aligned} |\mathring{R}_0^\epsilon (t)|_1 \le \frac{\delta }{2^7d}, \quad \text { for every } t \in [0,1]. \end{aligned}$$

Next, we decompose $\mathring{R}_0^\epsilon$ into basic directions. In order to stay within ${\mathcal {S}} _+$ of Lemma 2, we shift and normalise $\mathring{R}_0^\epsilon$ via

44 $$\begin{aligned}&{\mathrm{Id}}+ \frac{\mathring{R}_0^\epsilon (x,t)}{\varrho (x,t)}, \qquad \text {with} \nonumber \\&\varrho (x,t) := 2 \sqrt{\epsilon ^2 + |\mathring{R}_0^\epsilon (x,t)|^2} + \gamma _0(t), \end{aligned}$$

45 $$\begin{aligned}&\gamma _0(t) :=\frac{e(t) \left( 1-\frac{\delta }{2}\right) - \left( \int _{\mathbb {T}^{d}}|u_0|^2 (t) + 2 \int _0^t \int _{\mathbb {T}^{d}}\mathcal {A} (D u_0) D u_0 \right) }{d}. \end{aligned}$$

The role of $\sqrt{\epsilon ^2 + ..}$ is to avoid the degeneracy $|\mathring{R}_0(x,t)| =0$, whereas the role of $\gamma _0$ is to pump energy into the system, thus facilitating the step (11) $\rightarrow$ (14). Observe that $\gamma _0>0$ because of (11). The choice (44) yields in particular $\varrho \ge 2|\mathring{R}^\epsilon _0(x,t)|$ and hence

46 $$\begin{aligned} \frac{1}{2} {\mathrm{Id}}\le {\mathrm{Id}}+ \frac{\mathring{R}_0^\epsilon }{\varrho } \le \frac{3}{2} {\mathrm{Id}}. \end{aligned}$$

#### Remark 8

The set ${\mathcal {N}} \subset {\mathcal {S}} _+$ of Lemma 2 is fixed by (46) uniformly over the convex integration iterations.

Define

47 $$\begin{aligned} a_k(x,t) := \varrho ^\frac{1}{2} (x, t) \Gamma _k \left( {\mathrm{Id}}+ \frac{\mathring{R}_0^\epsilon (x,t)}{\varrho (x,t)}\right) . \end{aligned}$$

Thanks to Lemma 2 it holds

48 $$\begin{aligned} \varrho {\mathrm{Id}}+ \mathring{R}_0^\epsilon = \sum _{k \in K} \varrho \Gamma ^2_k \left( {\mathrm{Id}}+ \frac{\mathring{R}_0^\epsilon }{\varrho }\right) k \otimes k = \sum _{k \in K} a_k^2 \, k \otimes k. \end{aligned}$$

### Choice of $u_p$

Now we choose the principal corrector, motivated by the corrector $\tilde{u}$ that appeared in the initial part of Sect. 4. Let $W^k$ be the Mikado flow of Proposition 5 with $a_k$ defined by (47). Let

49 $$\begin{aligned} u_{p}(x,t) : = \sum _{k \in K} a_k (x,t) W^k (x,t). \end{aligned}$$

The disjoint supports of $W^k (t), W^{k'} (t)$, $k \ne k'$ imply

50 $$\begin{aligned} u_{p}\otimes u_{p}= \sum _{k \in K} a^2_k W^k \otimes W^k. \end{aligned}$$

Recall the notation $P_{\ne 0}f:= f - \fint _{\mathbb {T}^{d}}f$. Use (48) and (50) to write

51 $$\begin{aligned} \begin{aligned} u_{p}\otimes u_{p}- \mathring{R}_0^\epsilon&=\varrho {\mathrm{Id}}+ \sum _{k \in K} a^2_k \left( W^k \otimes W^k - k \otimes k \right) \\&= \varrho {\mathrm{Id}}+ \sum _{k \in K} a_k^2 P_{\ne 0} (W^k \otimes W^k ) + a_k^2 \left( \fint W^k \otimes W^k - k \otimes k \right) \end{aligned} \end{aligned}$$

We therefore have

52 $$\begin{aligned} \begin{aligned} \mathrm{div} \,(u_{p}\otimes u_{p}- \mathring{R}^\epsilon _0) =&\nabla \varrho + \sum _{k \in K} P_{\ne 0} (W^k \otimes W^k ) \nabla a_k^2 \\&+ \sum _{k \in K} \left( \fint W^k \otimes W^k - k \otimes k \right) \nabla a_k^2 + \sum _{k \in K} a_k^2 \mathrm{div} \,( W^k \otimes W^k). \end{aligned} \end{aligned}$$

#### Remark 9

Observe that for the original Mikados, or their concentrated version, the second line of (52) vanishes. These additional terms will be taken care of by (37) and (38).

In order to avoid troublesome solenoidality correctors of the last $\Sigma$ in (52), let us (Helmholtz) project it onto divergence free vectors by $P_H = Id - \nabla \Delta ^{-1} \mathrm{div} \,$ and balance the identity by incorporating $\nabla \Delta ^{-1} \mathrm{div} \,$ into the pressure, with the new pressure

$$\tilde{\varrho }:= \varrho + \Delta ^{-1} \mathrm{div} \,\sum _{k \in K} a^2_k \, \mathrm{div} \,\left( W^k \otimes W^k \right) .$$

Applying $P_{\ne 0}$ to both sides of the resulting identity, we arrive at

53 $$\begin{aligned} \begin{aligned}&\mathrm{div} \,(u_{p}\otimes u_{p}- \mathring{R}^\epsilon _0) = \nabla \tilde{\varrho }+ P_{\ne 0}\sum _{k \in K} P_{\ne 0} (W^k \otimes W^k ) \nabla a_k^2 \\&\quad + \,P_{\ne 0}\sum _{k \in K} \left( \fint W^k \otimes W^k - k \otimes k \right) \nabla a_k^2 + P_{\ne 0}P_H \sum _{k \in K} a_k^2 \mathrm{div} \,( W^k \otimes W^k). \end{aligned} \end{aligned}$$

### Choice of $u_c$

The corrector term $u_{c}$ has the following roles: (i) to cancel the highest-order bad term $`\mathrm{div} \,( W^k \otimes W^k)$’ of (53) via (38), (ii) to render the entire perturbation $u_p + u_c$ solenoidal and (iii) null-mean.

For (i), observe that (38) implies

54 $$\begin{aligned} P_{\ne 0}P_H \sum _{k \in K} a^2_k \partial _t Y^k+ P_{\ne 0}P_H \sum _{k \in K} a^2_k \mathrm{div} \,(W^k \otimes W^k) = 0. \end{aligned}$$

Thus taking

55 $$\begin{aligned} u_c^I := P_{\ne 0}P_H \sum _{k \in K} a^2_k Y^k \end{aligned}$$

will allow to cancel, with a part of the time derivative of $u_c^I$, the bad term $`\mathrm{div} \,( W^k \otimes W^k)$’ of (53).

For (ii), observe that thanks to $P_H$, $u_c^I$ is already solenoidal. Therefore it suffices to compensate lack of solenoidality of $u_{p}$. We will now define $u_c^{II}$ accordingly. By the definition (49) of $u_{p}$, the definition (34) of $W^k$, and since $\mathrm{div} \,\Psi _{\lambda _2}^k k =0$ (cf. the property (i) of (26)) we have

$$\begin{aligned} \mathrm{div} \,u_{p}(x,t)= & {} \sum _{k \in K} \mathrm{div} \,\left( a_k (x,t) \left( \Psi _{\lambda _2}^k \Phi ^k_{\mu , \lambda _1} k \right) (x-\omega t k) \right) \\= & {} \sum _{k \in K} \Psi ^k_{\lambda _2} (x) \; k \cdot \nabla \big ( a_k\Phi _{\mu , \lambda _1}^k(x - \omega t k) \big ). \end{aligned}$$

Therefore we define

56 $$\begin{aligned} u_c^{II}(t, \cdot ) := - \mathrm{div} \,\sum _{k \in K} {\mathcal {R}}^2_N \Big ( k \cdot \nabla \left( a_k (t) \Phi ^k_{\mu , \lambda _1}(\, \cdot \, - \omega t k ) \right) , \; \Psi ^k_{\lambda _2} \Big ), \end{aligned}$$

where ${\mathcal {R}}^2_N$ is the double antidivergence given by Proposition 4, and *N* will be fixed later (see the discussion at the beginning of Sect. 6).

Since $$\mathrm{div} \,\mathrm{div} \,{\mathcal {R}}^2_N = {\mathrm{Id}}\, P_{\ne 0}$$, $$\mathrm{div} \,u_c^{II} + \mathrm{div} \,u_{p}=0$$.

As $u_c^I, u_c^{II}$ are null-mean, to take into account the condition (iii), it suffices to define

57 $$\begin{aligned} u_{c}:= (u_c^I + u_c^{II}) - \fint u_p. \end{aligned}$$

### Reynolds stresses

Let us distribute $\partial _{t}(u_c^I + u_c^{II})$ and $\mathring{R}_0^\epsilon$ in (41) as follows

58 $$\begin{aligned} \begin{aligned}&\partial _{t}u_1 + \mathrm{div} \,(u_1\otimes u_1) - \mathrm{div} \,\mathcal {A} (Du_1 ) + \nabla \pi _1\\&\quad = \partial _{t}\left( P_{\ne 0}u_{p}+ u_c^{II} \right) + \mathrm{div} \,(u_0 \otimes u_{p}+ u_{p}\otimes u_0) \\&\qquad + \mathrm{div} \,(u_0 \otimes u_{c}+ u_{c}\otimes u_0 + u_{p}\otimes u_{c}+ u_{c}\otimes u_{p}+ u_{c}\otimes u_{c}) \\&\qquad + \partial _{t}u_c^{I} + \mathrm{div} \,(u_{p}\otimes u_{p}- \mathring{R}_0^\epsilon ) \\&\qquad + \mathrm{div} \,( \mathring{R}_0^\epsilon - \mathring{R}_0) \\&\qquad - \mathrm{div} \,\left( \mathcal {A} \big (D (u_0 + u_{p}+ u_{c}) \big ) - \mathcal {A} (D u_{0}) \right) - \nabla \pi _p \end{aligned} \end{aligned}$$

We rewrite the r.h.s. of (58) further, recasting it into a divergence form.

(i). First line of r.h.s. of (58)) The definition (49) of $u_{p}$, the definition (34) of $W^k$, and $0= k \cdot \nabla \Psi _{\lambda _2}^k (x)$ via property (i) of (26), give together

59 $$\begin{aligned} \partial _{t}u_{p}(t)= \sum _{k \in K} \Psi ^k_{\lambda _2}k \; \partial _{t}\left( a_k (\cdot , t) \Phi ^k_{\mu , \lambda _1} (\cdot - \omega k t) \right) . \end{aligned}$$

Using the above formula and the definition (56) of $u_c^{II}$, we define the antidivergence of the first line of r.h.s. of (58)

60 $$\begin{aligned} \begin{aligned} R_{lin}&:= \sum _{k \in K} {\mathcal {R}}_N \left( \partial _{t}\left( a_k (\cdot , t) \Phi ^k_{\mu , \lambda _1} (\cdot - \omega k t) \right) , \Psi ^k_{\lambda _2}k \right) \\&\quad - {\mathcal {R}}^2_N \left( k\cdot \nabla \partial _{t}\left( a_k (\cdot ,t) \Phi ^k_{\mu , \lambda _1} (\cdot - \omega k t \right) , \Psi ^k_{\lambda _2} \right) \\&\quad + (u_0 \otimes u_{p}+ u_{p}\otimes u_0). \end{aligned} \end{aligned}$$

(ii). Second line of r.h.s. of (58))

61 $$\begin{aligned} R_{corr} := u_0 \otimes u_{c}+ u_{c}\otimes u_0 + u_{p}\otimes u_{c}+ u_{c}\otimes u_{p}+ u_{c}\otimes u_{c}. \end{aligned}$$

(iii). Third line of r.h.s. of (58)) Here we use an important idea of [10]. Via the definition (55) of $u_c^I$ and the property (54) we have

$$\begin{aligned} \partial _{t}u_c^{I} =- P_{\ne 0}P_H \sum _{k \in K} a^2_k \, \mathrm{div} \,\left( W^k \otimes W^k \right) + P_{\ne 0}P_H \sum _{k \in K} (\partial _{t}a^2_k) Y^k. \end{aligned}$$

Adding the above identity to (53) that expresses $\mathrm{div} \,( u_{p}\otimes u_{p}- \mathring{R}^\epsilon _0)$, the $`\mathrm{div} \,( W^k \otimes W^k)$’ term cancel out and one has

$$\begin{aligned} \begin{aligned}&\partial _{t}u_c^{I} + \mathrm{div} \,(u_{p}\otimes u_{p}- \mathring{R}_0^\epsilon ) - \nabla \tilde{\varrho }\\&\quad =\sum _{k \in K} \left( W^k \otimes W^k - k \otimes k \right) \nabla a^2_k + P_{\ne 0}P_H \sum _{k \in K} (\partial _{t}a^2_k) Y^k. \end{aligned} \end{aligned}$$

Let us thus define, leaving out $\nabla \tilde{\varrho }$ since it will be accounted for by the pressure perturbation $q_p$,

62 $$\begin{aligned} R_{quadr}&:= \sum _{k \in K} \tilde{\mathcal {R}}_1 \left( \nabla a_k^2, P_{\ne 0}\left( W^k \otimes W^k \right) \right) + a_k^2 \Big ( \fint W^k \otimes W^k \!- k \otimes k \Big )\\&\quad + \mathrm {div}^{-1}P_{\ne 0}P_H \left( (\partial _{t}a^2_k) Y^k\right) , \end{aligned}$$

so that $$\mathrm{div} \,R_{quadr} = \partial _t u_c^I + \mathrm{div} \,(u_p \otimes u_p - \mathring{R}_0^\epsilon ) - \nabla \tilde{\varrho }.$$

(iv. Fourth line on r.h.s. of (58)) Let

63 $$\begin{aligned} R_{moll} := \mathring{R}_0^\epsilon - \mathring{R}_0. \end{aligned}$$

(v. Last line of r.h.s. of (58)) Let

64 $$\begin{aligned} R_{\mathcal {A} } := - \left( \mathcal {A} \big (D (u_0 + u_{p}+ u_{c}) \big ) - \mathcal {A} (D u_{0}) \right) . \end{aligned}$$

### Pressure

In order to balance for $\nabla \tilde{\varrho }$ and ensure null-trace of $\mathring{R}_1$, we choose $\pi _p$ such that

$$\begin{aligned} \nabla \pi _p= & {} \nabla \tilde{\varrho }+ \mathrm{div} \,\frac{1}{d} tr (R_{lin} + R_{corr} + R_{quadr} + R_{\mathcal {A} }) {\mathrm{Id}}, \qquad \text {i.e.} \\ \pi _p (x,t) + c (t):= & {} \tilde{\varrho }+\frac{1}{d} tr (R_{lin} + R_{corr} + R_{quadr} + R_{\mathcal {A} }), \end{aligned}$$

having freedom in choosing *c*(*t*), we set it so that $\pi _p (x,t)$ is null-mean.

### Conclusion

Comparing our choices (60), (61), (62,63), (64) and (65) for r.h.s. of (58) with its l.h.s. we have reached

65 $$\begin{aligned} \partial _{t}u_1 + \mathrm{div} \,(u_1\otimes u_1) - \mathrm{div} \,\mathcal {A} (Du_1 )+\nabla \pi _1 = -\mathrm{div} \,\mathring{R}_1 \end{aligned}$$

with

66 $$\begin{aligned} R_1 :=- (R_{lin} + R_{corr} + R_{quadr} + R_{moll} + R_{\mathcal {A} }). \end{aligned}$$

Notice that tensor $R_1$ is symmetric, since its components are symmetric (cf. respective definitions and Proposition 4).

## Estimates

We continue the proof of the main Proposition 1. In the previous section, given $(u_0, \pi _0, R_0)$ solving the non-Newtonian-Reynolds system, we defined $u_1 = u_0 + u_p + u_c$, $\pi _1$, and the new Reynolds stress $R_1$, required by Proposition 1. The perturbation $u_p$, the corrector $u_c$ and the error $R_1$ depend on the six parameters

67 $$\begin{aligned} \epsilon >0, \quad \mu , \lambda _1, \lambda _2, \omega \in \mathbb {N}, \quad N \in \mathbb {N}, \end{aligned}$$

which satisfy the condition (35). The mollification parameter $\epsilon$ helps to avoid degeneracies or singularities of $\mathcal {A}$. Let us immediately fix it so that

68 $$\begin{aligned} \epsilon \le \frac{\delta }{2^7 d}, \qquad |\mathring{R}_0^\epsilon (t) - \mathring{R}_0(t)|_1 \le \frac{\eta }{2} \quad \text { for every } t \in [0,1], \end{aligned}$$

where $\eta >0$ is the parameter appearing in the statement of the main Proposition 1.

In this section we estimate $u_p, u_c, R_1$ and the energy gap of the new solution $u_1$ in terms of the remaining five parameters $\mu , \lambda _1, \lambda _2, \omega , N$. They will be appropriately chosen in Sect. 7 so that (13a)–(14) hold, thus concluding the proof of the main Proposition 1.

### Remark 10

(Silent assumptions) For results of this section, we assume without writing it explicitly at each occasion: $(u_1, \pi _1, R_1)$ is the triple constructed in the previous section, the set of directions *K* is fixed by Remark 8, the relations (35) hold, the assumptions of Proposition 1 hold, the choice (69) holds.

### Constants

We distinguish two types of constants: the uniform ones (*M*’s) and the usual ones (*C*’s). None depend on $\mu ,\lambda _1, \lambda _2, \omega$.

More precisely, we denote by *M* any constant depending only on the following parameters

69 $$\begin{aligned} \begin{aligned}&\nu _0, \nu _1&\text {the parameters entering in the definition of the non-Newtonian tensor field} \mathcal {A} \\&q&\text {the exponent entering in the definition of the non-Newtonian tensor field } \mathcal {A} \\&e&\text {the energy profile fixed in the assumptions of Proposition 1} \\&\Phi , \Psi&\text {the profiles used in the definition of the Mikado functions in Sect. 4} \\&K&\text {the fixed set of directions, cf. Remark 8}. \end{aligned} \end{aligned}$$

Consequently, any universal constant *M* remains uniform over the convex integration iteration. We will not explicitly write the dependence of *M*’s on the objects in (70).

On the other side, we will denote by *C* (possibly with subscripts) any constant depending not only on the universal quantities (70), but also on

70 $$\begin{aligned} (u_0, \pi _0, R_0), \delta , \eta \qquad \text {given in the assumptions of Proposition 1}, \end{aligned}$$

71 $$\begin{aligned} \begin{aligned}&i,r&\text {if we are estimating the } W^{i,r} \text { norm} \\&N&\text {the (not yet fixed) parameter in (67)}, \end{aligned} \end{aligned}$$

Constants *C* will be controlled within each iteration step (i.e. in the proof of Proposition 1) by appropriate choices $\mu ,\lambda _1, \lambda _2, \omega$.

### Preliminary estimates: control of $a_k$

#### Proposition 6

Coefficients $a_k$ defined via (47) satisfy

72 $$\begin{aligned}&\left| a_k (t) \right| _2 \le 2 \delta ^\frac{1}{2}, \end{aligned}$$

73 $$\begin{aligned}&\qquad \qquad \quad \left| a_k\right| _{C^i_{x,t}} \le C_{i} \qquad \text { for } i \ge 0 \end{aligned}$$

#### Proof

The definition (45) of $\gamma _0$, assumption (11), and the assumed bounds on the energy profile $e \in [\frac{1}{2},1]$ yield $\gamma _0(t) \in [\frac{\delta }{8d},\delta ]$. This and the choice (69) of $\epsilon$ give, via the definition (44) of $\varrho$,

$$\begin{aligned} \varrho (x,t) \le 2 \epsilon + 2 |\mathring{R}_0^\epsilon (x,t)| + \gamma _0(t) \le 3 (|\mathring{R}_0^\epsilon (x,t)| + \delta ). \end{aligned}$$

Therefore, since $a^2_k = \varrho \Gamma ^2_k$ by its definition (47), whereas $\Gamma _k \le 1$ by Lemma 2,

$$\begin{aligned} |a_k(t)|_2^2 = \int _{{\mathbb {T}^{d}}} \varrho \Gamma ^2_k \le 3 (\delta + |\mathring{R}_0^\epsilon (t)|_1), \end{aligned}$$

The last inequality together with (43) yields (73).

Using smoothness of $\Gamma _k$ supported in the compact set (46) and that $\varrho \ge \gamma _0 \ge \frac{\delta }{8d}$, one has (74). $\square$

### Estimates for velocity increments

We will use now the improved Hölder inequality (18) and the preliminary estimates to control $u_p, u_c$.

#### Proposition 7

(Estimates for the principal increment $u_p$). For every $r \in [1,\infty ]$

74 $$\begin{aligned}&|u_{p}(t)|_r \le C_{r} \mu ^{\frac{d}{2} - \frac{d}{r}}, \end{aligned}$$

75 $$\begin{aligned}&|u_{p}(t)|_{W^{1,r}} \le C_{r} \lambda _2 \mu ^{\frac{d}{2} - \frac{d}{r}}. \end{aligned}$$

Moreover, there is a universal constant $M>0$ such that

76 $$\begin{aligned} |u_{p}(t)|_2 \le M \delta ^{1/2} + C \lambda _1^{-\frac{1}{2}}. \end{aligned}$$

#### Proof

The definition (49) of $u_{p}$ yields

77 $$\begin{aligned} |u_{p}(t)|_r \le \sum _{k \in K} |a_k (t) W^k (t)|_r. \end{aligned}$$

Using (74) to control $|a_k|_\infty$ and (36) to control $|W^k (t)|_r$, we obtain (75). An analogous computation gives (76).

Notice that for $$r=2$$ the power of $$\mu $$ in (75) is 0: for this reason, to reach (77), one needs more care. Recall from Proposition 5 that $$W^k$$ is $$\lambda _1$$-periodic. Therefore we may apply improved Hölder inequality (Proposition 3) to the r.h.s. of (78) and use (36) for $$ |W^k(t)|_2$$ to obtain

$$\begin{aligned} \begin{aligned} |u_{p}(t)|_2 \le M \sum _{k \in K} |a_k(t)|_2 + {\lambda _1}^{-\frac{1}{2}} |a_k(t)|_{C_x^1} \end{aligned} \end{aligned}$$

Let us now use (73), (74) to control $a_k$, reaching (77). $\square$

Now we deal with the corrector $u_c$. In order to shorten the related formulas, let us introduce

78 $$\begin{aligned} L(r) := \frac{ \mu ^{d-\frac{d}{r}}}{\omega } + \frac{\lambda _1 \mu }{\lambda _2} \mu ^{\frac{d}{2} - \frac{d}{r} } \left[ 1 + \lambda _2^2 \left( \frac{\lambda _1 \mu }{\lambda _2} \right) ^{N} \right] \end{aligned}$$

Observe that $r \mapsto L(r)$ is a non-decreasing map.

#### Proposition 8

(Estimates for the corrector $u_c$). For every $r \in (1, \infty )$ it holds

79 $$\begin{aligned} |u_{c}(t)|_{\dot{W}^{i,r}} \le C_{i,d,r,N} \lambda ^i_2 L(r). \end{aligned}$$

#### Proof

Recall that $u_{c}= (u_c^I + u_c^{II}) - \fint _{\mathbb {T}^{d}}u_p$ by its definition (57), with $u_{c}^I$ defined by (55) and $u_{c}^{II}$ defined by (56).

The Calderón-Zygmund estimate, (74), and (36) give

80 $$\begin{aligned} |u_{c}^I (t)|_{\dot{W}^{i,r}} \le C_{r} \frac{\lambda ^i_2 \mu ^{d - \frac{d}{r}}}{\omega } \end{aligned}$$

For the estimate of $u_c^{II}$ in $\dot{W}^{i,r}$ we use the inequality (23) for $j=i+1$, $s=\infty$, which yields

$$\begin{aligned}&| u_c^{II}|_{\dot{W}^{i,r}} \le C_{j,d,r,N} \lambda _2^{i+1}\\&\quad \times \left( \frac{1}{\lambda _2^{2}} | \nabla ( a_k \Phi ^k_{\mu , \lambda _1})|_{r} + \frac{1}{\lambda _2^{N}} |\nabla ^{N+1}( a_k \Phi ^k_{\mu , \lambda _1})|_{r} + \frac{1}{\lambda _2^{2N+i+1}} |\nabla ^{2N +i+2}( a_k \Phi ^k_{\mu , \lambda _1})|_{r} \right) . \end{aligned}$$

Now we estimate $a_k$ by (74) and $\Psi ^k_{\mu , \lambda _1}$ by (32). Using the assumption (35), we arrive at

81 $$\begin{aligned} \begin{aligned} | u_c^{II}|_{\dot{W}^{i,r}} \le C_{i,d,r,N} {\lambda ^i_2} \frac{\lambda _1 \mu }{{\lambda _2}} \mu ^{\frac{d}{2} -\frac{d}{r}} \left[ 1+ \lambda _2^2 \left( \frac{\lambda _1 \mu }{{\lambda _2}} \right) ^{N} \right] . \end{aligned} \end{aligned}$$

Recall $u_{c}= (u_c^I + u_c^{II}) - \fint _{\mathbb {T}^{d}}u_p$ by its definition (57). Therefore, putting together (81), (82), and

$$\begin{aligned} \Big | \fint u_p \Big | \le \frac{\lambda _1 \mu }{{\lambda _2}} \mu ^{-\frac{d}{2}} \le \frac{\lambda _1 \mu }{\lambda _2} \mu ^{\frac{d}{2} - \frac{d}{r} } \end{aligned}$$

valid via (17) with $r=1$ and $v = \Psi ^k$, yields (80). $\square$

### Estimates on the Reynolds stress

Recall (67)

$$\begin{aligned} R_1 :=- (R_{lin} + R_{corr} + R_{quadr} + R_{moll} + R_{\mathcal {A} }). \end{aligned}$$

In this section we estimate each term of $R_1$. For our further purposes $L^1$ estimates suffice, but due to using Calderón-Zygmund theory, some estimates are phrased as $L^r$ ones.

#### Proposition 9

(Estimates on the principal Reynolds $R_{quadr}$). For every $r \in (1, \infty )$, it holds

82 $$\begin{aligned} |R_{quadr}(t)|_r \le C_{r} \left( (\omega ^{-1} + \lambda ^{-1}_1 ) \mu ^{d-\frac{d}{r}} + \lambda _2^{-1} \lambda _1 \mu \right) \end{aligned}$$

#### Proof

Recall the definition (62,63) of $R_{quadr}$. Let us estimate its three terms in order of their appearance.

(i) The first term of $R_{quadr}$ is the sum over *k* of $\tilde{\mathcal {R}}_1 (\nabla a_k^2, P_{\ne 0}(W^k\otimes W^k))$. The term $P_{\ne 0}(W^k\otimes W^k))$ is null mean and it oscillates at the frequency $\lambda _1$, since $W^k$ does. Therefore (21) with (74) and (36) give

83 $$\begin{aligned} |\tilde{\mathcal {R}}_1 (\nabla a_k^2, P_{\ne 0}(W^k \otimes W^k))|_r \le C_{r} \lambda ^{-1}_1 |P_{\ne 0}(W^k \otimes W^k))|_r |\nabla ^2 (a_k^2)|_\infty \le C_{r} \lambda ^{-1}_1\mu ^{ d - \frac{d}{r}}. \end{aligned}$$

(ii) The second term of $R_{quadr}$ is the sum over *k* of $a_k^2 ( \fint W^k\otimes W^k - k \otimes k)$. We use estimate (74) to control $a_k$ terms and (37) to control the Mikado terms

84 $$\begin{aligned} \Big | a_k^2 \Big ( \fint W^k\otimes W^k- k \otimes k \Big ) \Big |_r \le |a^2_k|_r \Big | \fint W^k \otimes W^k - k \otimes k \Big | \le C_r \frac{\lambda _1 \mu }{\lambda _2}. \end{aligned}$$

(iii) The last term is the sum over *k* of $\mathrm {div}^{-1}P_H \left( P_{\ne 0}(\partial _{t}a^2_k) Y^k \right)$. We deal with $\mathrm {div}^{-1}$ via (19) and with $P_H$ via Calderón-Zygmund, control $\partial _{t}a_k$ using (74), and use (36) to estimate $Y^k$. Hence

85 $$\begin{aligned} \left| \mathrm {div}^{-1}P_H \left( P_{\ne 0}(\partial _{t}a^2_k) Y^k \right) \right| _r (t) \le C_r \left| P_{\ne 0}(\partial _{t}a^2_k) Y^k\right| _r (t) \le C_{r} |Y^k(t)|_r \le C_{r} \frac{ \mu ^{d-\frac{d}{r}}}{\omega }. \end{aligned}$$

Together, (84), (85), (86) yield (83). $$\square $$

#### Proposition 10

(Estimate on $R_{lin}$). It holds

86 $$\begin{aligned} |R_{lin}|_1 (t) \le C_{N} \bigg [ \mu ^{-\frac{d}{2}} + \frac{ \lambda _1 \mu ^{1-\frac{d}{2}} \omega }{\lambda _2} \Big ( 1 + \lambda _2^2 \Big (\frac{\lambda _1 \mu }{\lambda _2} \Big )^{N} \Big ) \bigg ] . \end{aligned}$$

#### Proof

Recall the definition (60) of $R_{lin}$. It involves three terms, which we estimate in order of their appearance.

(i) The first term of $R_{lin}$ is the sum over *k* of

$${\mathcal {R}}_N \big ( (\partial _{t}a_k) \Phi ^k_{\mu , \lambda _1} + \omega a_k k \cdot \nabla \Phi ^k_{\mu , \lambda _1}, \Psi ^k_{\lambda _2} k \big ).$$

Using (21) with $|u|_s = |\Psi ^k|_\infty$, the assumption (35), and disposing of $a_k$ as usual, one has

87 $$\begin{aligned} | {\mathcal {R}}_N \left( \partial _{t}\left( a_k (\cdot , t) \Phi ^k_{\mu , \lambda _1} (\cdot - \omega k t) \right) , \Psi ^k_{\lambda _2}k \right) |_1 (t) \le C_{d, N} \frac{\omega \lambda _1 \mu ^{1 - d/2}}{\lambda _2} \Big [ 1 + \lambda _2 \Big ( \frac{\lambda _1 \mu }{\lambda _2} \Big )^N \Big ]. \end{aligned}$$

(ii) The second term of $R_{lin}$ is the sum over *k* of

$$\begin{aligned} {\mathcal {R}}^2_N (k \cdot \nabla \partial _{t}( a_k \Phi ^k_{\mu , \lambda _1} (\, \cdot \, - \omega k t ), \Psi ^k_{\lambda _2}). \end{aligned}$$

We observe that

88 $$\begin{aligned}&|\nabla ^{i+1} \partial _{t}\left( a_k \Phi ^k_{\mu , \lambda _1} (\, \cdot \, - \omega k t ) \right) |_1 \le |\partial _t a_k \Phi ^k_{\mu , \lambda _1}|_{W^{i+1,1}} + \omega |k| | a_k \nabla \Phi ^k_{\mu , \lambda _1}|_{W^{i+1,1}} \nonumber \\&\quad \le C \omega \lambda _1^{i+2} \mu ^{i+2 - \frac{d}{2}}. \end{aligned}$$

Using the computation (89) in (23) with $j=0$ and $|u|_s = |\Psi ^k|_\infty$, we get

89 $$\begin{aligned} \big | {\mathcal {R}}^2_N \left( k \cdot \nabla \partial _{t}\left( a_k \Phi ^k_{\mu , \lambda _1} (\, \cdot \, - \omega k t ) \right) , \Psi ^k_{\lambda _2} \right) \big |_1 \le C_{d,N} \, \omega \mu ^{-\frac{d}{2}} \left( \frac{\lambda _1 \mu }{\lambda _2} \right) ^2 \left[ 1 + \lambda _2^2 \Big ( \frac{\lambda _1 \mu }{\lambda _2} \Big )^N \right] .\nonumber \\s \end{aligned}$$

(iii) The third term of $R_{lin}$ equals $u_0 \otimes u_{p}+ u_{p}\otimes u_0$, so we write using (75)

90 $$\begin{aligned} |u_0|_\infty |u_{p}|_1 \le C\mu ^{-\frac{d}{2}}. \end{aligned}$$

Putting together (88), (90), (91), and observing that the right-hand sides of both (88) and (90) are estimated by $C_{d,N} \, \omega \mu ^{-\frac{d}{2}} \frac{\lambda _1 \mu }{\lambda _2} [ 1 + \lambda _2^2 ( \frac{\lambda _1 \mu }{\lambda _2})^N]$ thanks to (35), one has (87). $\square$

#### Proposition 11

(Estimates on $R_{corr}$). Let *L*(2) be given by (79). It holds

91 $$\begin{aligned} \begin{aligned} |R_{corr} (t)|_1 \le C_{N} \big (L(2) + L^2(2) \big ). \end{aligned} \end{aligned}$$

#### Proof

By the definition (61) of $R_{corr}$ we have

$$\begin{aligned} |R_{corr}|_1 (t) \le C(|u_0|_2 |u_{c}|_2 + |u_{p}|_{2} |u_{c}|_{2} + |u_{c}|^2_{2})(t), \end{aligned}$$

since $|u_{p}|_{2} \le C$ via (75) and $|u_{c}|_{2} \le C_N L(2)$ via (80), we have (92). $\square$

#### Proposition 12

(Estimates on the dissipative Reynolds $R_{\mathcal {A} }$). For $q \in (1, \infty )$ being the growth parameter of $\mathcal {A}$ it holds

92 $$\begin{aligned} |R_{\mathcal {A} }(t)|_r \le \left\{ \begin{aligned}&C \big ( {( \lambda _2 \mu ^{\frac{d}{2} - \frac{d}{r}})^{q-1}} + (\lambda _2 L(r))^{q-1} \big )&\text { for } \nu _0=0, q { \le } 2, \;&\text {any } r>1, \\&C \big ( { \lambda _2 \mu ^{\frac{d}{2} - \frac{d}{r}}} + \lambda _2 L(r) \big )&\text { for } \nu _0>0, q {\le } 2, \;&\text {any } r>1, \\&C \Big ( { \lambda _2 \mu ^{\frac{d}{2} - \frac{d}{r(q-1)}}} + { \big ( \lambda _2 \mu ^{\frac{d}{2} - \frac{d}{r(q-1)}} \big )^{q-1}} \\&\qquad + \lambda _2 L(r(q-1)) + (\lambda _2 L(r(q-1)))^{q-1} \Big )&\text { for } q\ge 2, \;&\text {any } r(q-1)>1. \end{aligned} \right. \end{aligned}$$

#### Proof

By definition (65), we have

$$|R_{\mathcal {A} }| = |\mathcal {A} (Du_0 + Du_p + Du_c) - \mathcal {A} (Du_0)|.$$

Therefore the inequality (15) gives the pointwise estimate

$$\begin{aligned} |R_{\mathcal {A} }| \le \left\{ \begin{aligned}&C_{\nu _1} |Du_p + Du_c|^{q-1}&\text { for } \nu _0=0, q\le 2 \\&C_{\nu _0} |Du_p + Du_c|&\text { for } \nu _0>0, q\le 2 \\&C_{q, \nu _0, \nu _1} |Du_p + Du_c| \left( 1 + |Du_0|^{q-2} + |Du_1|^{q-2} \right)&\text { for } q\ge 2 \end{aligned} \right. \end{aligned}$$

Using Jensen inequality and $\frac{1}{q-1} {\ge } 1$ in the first case, and Hölder inequality with $q-1$, $\frac{q-1}{q-2}$ in the last case, one has

$$\begin{aligned} |R_\mathcal {A} |_r \le \left\{ \begin{aligned}&C |D u_p + Du_c|_r^{q-1}&\text { for } \nu _0=0, q {\le } 2 \\&C |Du_p + Du_c|_r&\text { for } \nu _0>0, q {\le } 2 \\&C |Du_p + Du_c|_{r(q-1)} \left( 1 + |Du_0|_{r(q-1)}^{q-2} + |Du_1|_{r(q-1)}^{q-2} \right)&\text { for } q\ge 2 \end{aligned} \right. \end{aligned}$$

For any $s \in (1, \infty )$ the estimate (80) controls $|D u_c|_s$ via $\lambda _2 L(s)$, whereas $\lambda _2 \mu ^{d/2 - d/s}$ controls $|D u_p|_s$ thanks to (76). This closes the case $q<2$ of (93). Recalling that $u_1 = u_0 +u_p + u_c$ and that *C* may contain norms of $u_0$, we obtain the case $q\ge 2$. $\square$

#### Remark 11

For the current purpose of proving Proposition 1 and thus Theorem A, the case $q \le 2$ of (93) suffices. We included already the case $q\ge 2$, because it is needed to prove Theorem B.

Immediately from the definition of $R_{moll}$ in (64) and the choice of $\epsilon$ in (69) we have

#### Proposition 13

(Estimate on $R_{moll}$). It holds

93 $$\begin{aligned} |R_{moll}(t)|_1 \le \eta /2 \end{aligned}$$

where $\eta$ is the parameter appearing in the assumptions of Proposition 1.

### Estimates on the energy increment

We intend to approach the desired energy profile *e*(*t*), i.e. perform the step (11) $\rightarrow$ (14). Let us thence define $\delta E$ as follows

94 $$\begin{aligned} \delta E (t):= \Bigg |e(t) \Bigg (1 - \frac{\delta }{2} \Bigg ) - \left( \int |u_1|^2 (t) + 2 \int _0^t \int \mathcal {A} (D u_1) D u_1 \right) \Bigg |. \end{aligned}$$

Recall quantities *L* of (79). We will show

#### Proposition 14

(energy iterate). For $q \in (1, \infty )$ being the growth parameter of $\mathcal {A}$ it holds

95 $$\begin{aligned} \delta E(t)\le & {} \frac{\delta }{16} e(t) + C_{N} \left( \lambda ^{-1}_1 + {\mu ^{-\frac{d}{2}}} + L(2) + L(2)^2 + { \lambda _2 \mu ^{\frac{d}{2} - \frac{d}{q}} + \left( \lambda _2 \mu ^{\frac{d}{2} - \frac{d}{q}} \right) ^q }\right. \nonumber \\&\left. + \lambda _2 L(q) + \big ( \lambda _2 L(q) \big )^q \right) . \end{aligned}$$

#### Proof

Recall (51). Taking its trace and recalling that $\mathring{R}_0^\epsilon$ is traceless we have

96 $$\begin{aligned} |u_{p}|^2 = d \varrho +\sum _{k \in K} a^2_k P_{\ne 0}|W^k|^2+ a_k^2 \left( \fint |W^k|^2 - |k|^2\right) . \end{aligned}$$

By the definition (44) it holds $d\varrho = 2 d \sqrt{\epsilon ^2 + |\mathring{R}_0^\epsilon |^2} + d\gamma _0$, therefore

$$\begin{aligned} |u_{p}|^2 -d \gamma _0 = 2d\sqrt{\epsilon ^2 + |\mathring{R}_0^\epsilon |^2}+\sum _{k \in K} a^2_k P_{\ne 0}|W^k|^2 + a_k^2 \left( \fint |W^k|^2 - |k|^2 \right) \end{aligned}$$

Integrating and using $\sqrt{\epsilon ^2 +|x|^2} \le \epsilon +|x|$, we have

97 $$\begin{aligned} \left| \int |u_{p}|^2 - d \gamma _0 \right| \le 2d \epsilon + 2d |\mathring{R}^\epsilon _0(t)|_1 +\sum _{k \in K} \Bigg | \int a^2_k P_{\ne 0}|W^k|^2 \Bigg | + |a_k|_2^2 \Bigg | \fint |W^k|^2 - |k|^2 \Bigg | \end{aligned}$$

We estimate the first two terms of the r.h.s. of (98) using (69) and (43) as follows

$$\begin{aligned} 2d \epsilon + 2d |\mathring{R}^\epsilon _0(t)|_1 \le \frac{\delta }{2^6} + \frac{\delta }{2^6} \le \frac{\delta }{2^4} e(t), \end{aligned}$$

where in the second inequality we used the assumption $e(t) \ge \frac{1}{2}$. This in (98) yields

98 $$\begin{aligned} \Bigg | \int |u_{p}|^2 - d \gamma _0 \Bigg | \le \frac{\delta }{2^4}e(t) +\sum _{k \in K} \Bigg | \int a^2_k P_{\ne 0}|W^k|^2 \Bigg | + |a_k|_2^2 \Bigg | \fint |W^k|^2 - |k|^2 \Bigg | \end{aligned}$$

The first integral of r.h.s. of (99) involves a $\lambda _1$-oscillating function $P_{\ne 0}|W^k|^2$, recall Proposition 5. Therefore, using (17), then (74) to control $a_k$ and (36) for $W^k$, we have

99 $$\begin{aligned} \Bigg | \int a^2_k P_{\ne 0}|W^k|^2 \Bigg | \le C \lambda _1^{-1} |a^2_k|_\infty |(P_{\ne 0}|W^k|)|^2_2 \le C \lambda _1^{-1}. \end{aligned}$$

For the integral following the second sum in (99), we use (37) and (74) to get

100 $$\begin{aligned} |a_k|_2^2 \Bigg | \fint |W^k|^2 - |k|^2 \Bigg | \le C \frac{\lambda _1 \mu }{\lambda _2}. \end{aligned}$$

We plug (100) and (101) to (99) and obtain

101 $$\begin{aligned} \Bigg | \int |u_{p}|^2 - d \gamma _0 \Bigg | \le \frac{\delta }{2^4} e(t) + C \Bigg ( \frac{1}{\lambda _1} + \frac{\lambda _1 \mu }{\lambda _2} \Bigg ). \end{aligned}$$

Use $u_1 = u_0 + u_p + u_c$ in the definition (95) of *E* to write for the time instant *t*

$$\begin{aligned} \delta E(t)\le & {} \Bigg | \int |u_p|^2 - d \gamma _0 \Bigg |+ \Bigg | \int |u_c|^2 + 2 (u_0 u_c + u_0 u_p + u_p u_c) \Bigg | \\&+ 2 \Bigg |\int _0^t \int \mathcal {A} (D u_1) D u_1 - \mathcal {A} (D u_0) D u_0 \Bigg |, \end{aligned}$$

where a cancellation occurs, thanks to the definition (45) of $\gamma _0$. Inequality (102) allows to control the first term of the r.h.s. above. For the last term we use (16), next Hölder inequality with *q*, $\frac{q}{q-1}$, and finally $u_1 = u_0 +u_p + u_c$ to get

$$\begin{aligned} \int | \mathcal {A} (D u_1) D u_1 - \mathcal {A} (D u_0) D u_0| \le C \left( |Du_p + Du_c|_q + |Du_p + Du_c|_q^q \right) . \end{aligned}$$

Thus, integrating in time over $[0,t] \subseteq [0,1]$,

$$\begin{aligned}&\int _0^t \int |\mathcal {A} (D u_1) D u_1 - \mathcal {A} (D u_0) D u_0|\\&\quad \le C \sup _{\tau \in [0,1]} \left( |Du_p(\tau ) + Du_c(\tau )|_q + |Du_p(\tau ) + Du_c(\tau )|_q^q \right) . \end{aligned}$$

Consequently

$$\begin{aligned} \begin{aligned} \delta E(t)&\le \frac{\delta }{2^4} e(t) + C \left( \frac{1}{\lambda _1} + \frac{\lambda _1 \mu }{\lambda _2} \right) \\&\quad + C \left( |u_c|_2^2 + |u_0|_2 |u_c|_2 + |u_0|_\infty |u_p|_1 + |u_c|_{2} |u_p|_2 \right) (t) \\&\quad + C \sup _{\tau \in [0,1]} \left( |Du_p(\tau ) + Du_c(\tau )|_q + |Du_p(\tau ) + Du_c(\tau )|_q^q \right) . \end{aligned} \end{aligned}$$

The terms in the second line above are estimated, using (80) for $u_c$ and (75) for $u_p$, by $C_{N} ( L(2)^2 + L(2) + \mu ^{-d/2})$. Observe that *L*(2) of this term can absorb $\lambda _2^{-1} \lambda _1 \mu$ of the first line. The terms in the last line are estimated by ${ \lambda _2 \mu ^{\frac{d}{2} - \frac{d}{q}} + \big ( \lambda _2 \mu ^{\frac{d}{2} - \frac{d}{q}} \big )^q } + \lambda _2 L(q) + (\lambda _2 L(q))^q$, using (76) for $Du_p$, (80) for $Du_c$. We thus arrived at (96). $\square$

## Proof of the Main Proposition 1

Having at hand the estimates of the previous section, we are ready to show that $(u_1, q_1, R_1)$ constructed in Sect. 5 satisfy the inequalities (13a)–(14).

The estimates of the previous section have at their right-hand sides two type of terms: ones where the parameters $\lambda _2, \lambda _1$, $\mu$, $\omega$ are intertwined, and the remaining ones. These remaining ones can be made small simply by choosing the relevant parameters large. The terms with $\lambda _2, \lambda _1$, $\mu$, $\omega$ interrelated need more care, so let us focus on them. They contain two little technical nuisances: (i) appearance of *N* and (ii) estimates for some parts of *R* not holding in $L^1$. Let us ignore these nuisances for a moment, which is easily acceptable after recalling (i) ${\mathcal {R}}_\infty$ of Remark 6 (which heuristically cancels the terms involving *N*) and that (ii) estimates for *R* hold in $L^{r}$ for any $r>1$, whereas an $\epsilon$ of room is assured by the assumed sharp inequality $q<\frac{2d}{d+2}$. So for a moment let us consider estimates of Sect. 6 allowing for $|R|_1$ and disregarding the terms with *N*. After inspection, we see that smallness of their right-hand sides where $\lambda _2, \lambda _1$, $\mu$, $\omega$ are intertwined, needed for Proposition 1 is precisely the smallness of (40), Remark 7. Therefore we will proceed as follows.

Firstly, guided by (40), we will choose relation between magnitudes of $\lambda _2, \lambda _1$, $\mu$, $\omega$. To this end we postulate

102 $$\begin{aligned} \lambda _1 := \lambda , \quad \mu := \lambda ^a, \quad \omega := \lambda ^b, \quad \lambda _2 := \lambda ^c, \end{aligned}$$

and choosing relation between magnitudes means picking *a*, *b*, *c* so that (40) are strictly decreasing in $\lambda$.

Secondly, we will need to make sure that when $r>1$ and *N* appear, the relations between magnitudes do not change. This will be achieved by choosing *N* large and *r* small in relation to *a*, *b*, *c*.

Finally, we will send $\lambda \rightarrow \infty$ to reach (13a)–(14).

### Picking magnitudes *a*, *b*, *c*

The requirement that powers in (40) rewritten in terms of (103) are negative reads

103 $$\begin{aligned} \begin{aligned} \frac{\lambda _1 \mu }{\lambda _2}&= \lambda ^{1+a-c}&i.e.&1+a-c&< 0, \\ \frac{\lambda _1 \mu }{\lambda _2} \, \cdot \, \frac{\omega }{\mu ^{d/2}}&= \lambda ^{1+(1 - \frac{d}{2})a-c + b}&i.e.&1+ \Big (1 - \frac{d}{2}\Big )a-c + b&< 0, \\ \frac{\mu ^{d/2}}{\omega }&= \lambda ^{ad/2-b}&i.e.&\frac{d}{2}a -b&< 0, \\ \lambda _2 \mu ^{\frac{d}{2} - \frac{d}{q}}&= \lambda ^{c + (\frac{d}{2} - \frac{d}{q})a}&i.e.&c - \Big ( \frac{d}{q} -\frac{d}{2} \Big )a&< 0. \end{aligned} \end{aligned}$$

These conditions on *a*, *b*, *c* can be simultaneously achieved as follows.

1. The conditions not involving *b* amount to the requirement
  104 $$\begin{aligned} 1+ a< c< \Big ( \frac{d}{q} -\frac{d}{2} \Big )a. \end{aligned}$$
  From the assumption $q < \frac{2d}{d+2}$ of Proposition 1 it follows that $d/q - d/2 > 1$. Therefore satisfying (105) is possible with *a* large. More precisely, let us pick
  105 $$\begin{aligned} a > \frac{3}{d \big ( \frac{1}{q} - \frac{d+2}{2d} \big )}. \end{aligned}$$
  Then between $1 + a$ and $(d/q - d/2)a$ there are at least two natural numbers. We then fix $c \in \mathbb {N}$ as the largest natural number satisfying (105). Notice that there is still at least one natural number between $1+a$ and *c*.
2. Let us fix $b \in \mathbb {N}$ so that
  106 $$\begin{aligned} \frac{d}{2}a< b< \Big ( \frac{d}{2} - 1 \Big ) a + c -1. \end{aligned}$$
  This is possible, because, as observed in point (1), there is at least one natural number between $1+a$ and *c* and thus also between *ad*/2 and $(d/2-1)a + c -1$. The condition (107) automatically verifies the two conditions concerning *b*.

Let us denote by $-\zeta <0$ the largest power of those appearing in (104). We have just showed

107 $$\begin{aligned} \frac{\mu ^{d/2}}{\omega } \le \lambda ^{-\zeta }, \quad \lambda _2 \frac{\mu ^{d-\frac{d}{q}}}{\omega } \le \lambda ^{-\zeta }, \quad \frac{\lambda _1 \mu }{{\lambda _2}} \le \lambda ^{-\zeta }, \quad {\lambda _2} \mu ^{\frac{d}{2} - \frac{d}{q}} \le \lambda ^{-\zeta }, \quad \frac{\lambda _1 \mu }{{\lambda _2}} \cdot \frac{\omega }{\mu ^{d/2}} \le \lambda ^{-\zeta }. \end{aligned}$$

### Fixing *N* and $r_0>1$

Let us fix $N \in \mathbb {N}$ so that

108 $$\begin{aligned} c - \zeta N \le 0. \end{aligned}$$

This choice yields

109 $$\begin{aligned} 1 + \lambda _2^2 \left( \frac{\lambda _1 \mu }{\lambda _2} \right) ^N \le 1 + \lambda ^{2c - 2 \zeta N} \le 2. \end{aligned}$$

Using the definition (79) of *L* with (108) and (110), one has

110 $$\begin{aligned} L(q) \le C\lambda ^{-\zeta }, \quad L(2) \le C\lambda ^{-\zeta }, \quad \lambda _2 L(q) \le C\lambda ^{-\zeta }. \end{aligned}$$

Importantly, fixing the gauge *N* freezes all $C_N$’s in estimates to *C*.

Let us fix also an exponent $r_0 \in (1, \infty )$ (close to 1) such that

111 $$\begin{aligned} \begin{aligned} \left( d - \frac{d}{r_0} \right) a \le \frac{1}{2}. \end{aligned} \end{aligned}$$

This is possible because the l.h.s. above vanishes as $r_0 \rightarrow 1$.

### Obtaining (13a)–(14)

Recall that $\delta , \eta$ are given small numbers. Since $u_1-u_0 = u_{p}+ u_{c}$, we have by (77) and (80)

112 $$\begin{aligned} |(u_1-u_0)(t)|_{L^2} \le M \delta ^\frac{1}{2} + C \big ( \lambda _1^{-1/2} + L(2) \big ) \le M \delta ^\frac{1}{2} + C \big ( \lambda ^{-1/2} + \lambda ^{-\zeta } \big ), \end{aligned}$$

recalling for the latter inequality that $\lambda _1 = \lambda$ via (103), and (111). Choose $\lambda \in \mathbb {N}$ large in relation to *C* we thus have

$$\begin{aligned} |(u_1-u_0)(t)|_{L^2} \le 2M \delta ^\frac{1}{2} = M_0 \delta ^\frac{1}{2}, \end{aligned}$$

defining $M_0 := \frac{M}{2}$, hence (13a). Notice that $M_0$ depends only on the universal constant *M*. Thus $M_0$ itself is universal, i.e. it may depend on the quantities (70), but not on the quantities (71).

Similarly to obtaining (113), using (76), (79) and (80) we have

113 $$\begin{aligned} |u_1 - u_0|_{W^{1,q}} \le C \big ( \lambda _2 \mu ^{d/2 - d/q} + L(q) + \lambda _2 L(q) \big ) \le C \lambda ^{-\zeta }, \end{aligned}$$

where for the term $\lambda _2 \mu ^{d/2 - d/q}$ we used (108). Estimate (13b) follows by choosing $\lambda$ big enough.

Recall that $R_1 = - (R_{lin} + R_{corr} + R_{quadr} + R_{moll} + R_{\mathcal {A} })$ by its definition (67). By (87) we have, with $C_N$ now fixed to *C* by the choice (109) of *N*

114 $$\begin{aligned} |R_{lin}|_1 (t) \le C \bigg [ \mu ^{-\frac{d}{2}} + \frac{ \lambda _1 \mu ^{1-\frac{d}{2}} \omega }{\lambda _2} \Big ( 1 + \lambda _2^2 \Big (\frac{\lambda _1 \mu }{\lambda _2} \Big )^{N} \Big ) \bigg ] \le C(\lambda ^{-ad/2} + \lambda ^{-\zeta } 2), \end{aligned}$$

where for the second inequality we invoked $\mu = \lambda ^a$ by (103), (108), and (110).

Similarly, using (92) and (111)

115 $$\begin{aligned} |R_{corr} (t)|_1 \le C \lambda ^{-\zeta }. \end{aligned}$$

For the $L^1$-estimate of $R_{quadr}$ we need to switch to the $L^{r_0}$ estimate, where $r_0$ was fixed in (112). We have, using (83)

$$\begin{aligned}&|R_{quadr}(t)|_1 \le |R_{quadr}(t)|_{r_0} \le C ( \omega ^{-1} + \lambda ^{-1}_1) \mu ^{d-\frac{d}{r_0}} + C \lambda ^{-1} \lambda _1 \mu \nonumber \\&\quad \le C( \lambda ^{(d-d/r_0)a - b} +\lambda ^{(d-d/r_0)a - 1} ) + C \lambda ^{-\zeta }. \end{aligned}$$

Thanks to the choice of $r_0$ in (112), we hence have

116 $$\begin{aligned} |R_{quadr}(t)|_1 \le C\lambda ^{-\frac{1}{2}} + C \lambda ^{-\zeta }. \end{aligned}$$

Similarly, for the estimate of $R_\mathcal {A}$ we use (93) with $q \in (1,2)$, obtaining

$$\begin{aligned} |R_\mathcal {A} (t)|_1 \le |R_{\mathcal {A} }(t)|_q \le \left\{ \begin{aligned}&C \Big ( { \big (\lambda _2 \mu ^{\frac{d}{2} - \frac{d}{q}} \big )^{q-1}} + \lambda _2 L(q) \Big )^{q-1}&\text { for } \nu _0=0, q<2,\\&C \big ( { \lambda _2 \mu ^{\frac{d}{2} - \frac{d}{q}} } + \lambda _2 L(q) \big )&\text { for } \nu _0>0, q<2. \end{aligned} \right. \end{aligned}$$

Therefore by (108), (111) and $q-1 \in (0,1)$

117 $$\begin{aligned} |R_\mathcal {A} (t)|_1 \le C \lambda ^{-\zeta (q-1)}. \end{aligned}$$

Together, the terms $R_{lin}, R_{corr}, R_{quadr}, R_\mathcal {A}$ are bounded in view of, respectively, (115), (116), (117), and (118) by $C \lambda ^{-\zeta '}$ with certain $\zeta '>0$:

$$\begin{aligned} |R_{lin}(t)|_1 + |R_{corr}(t)|_1 + |R_{quadr}(t)|_1 + |R_\mathcal {A} (t)|_1 \le C \lambda ^{-\zeta '} \end{aligned}$$

Therefore, using for the remaining $R_{moll}$ the estimate (94), we have

118 $$\begin{aligned} |R_1(t)|_1 \le \frac{\eta }{2} + C \lambda ^{-\zeta '}. \end{aligned}$$

thus showing (13c) by taking $\lambda$ large.

Let us show the last remaining inequality (14). By (96), with $C_N$ fixed to *C* by the choice (109) of *N*, we have in view of (108) and (111)

119 $$\begin{aligned} \delta E(t) \le \frac{\delta }{16} e(t) + C \left( \lambda ^{-1} + {\lambda ^{-\frac{ad}{2}}} + \lambda ^{-\zeta } + \lambda ^{-2\zeta } + \lambda ^{-\zeta } + \lambda ^{-q\zeta } \right) \le \frac{\delta }{16} e(t) + \frac{\delta }{32} \le \frac{\delta }{8} e(t) \end{aligned}$$

The proof of Proposition 1 is concluded.

## Proof of Theorem A

We will iterate Proposition 1. Let us start at the trivial solution $(u_0, \pi _0, R_0) \equiv 0$ with $\delta _0 =1$. At the *n*th step we take $\delta _n := {2^{-n}}$ and $\eta _n := \frac{\delta _{n+1}}{2^8 d}$, hence $|R_{n+1} (t)|_{L^1} \le \eta _{n+1}= \frac{1}{2} \frac{\delta _{n+1}}{2^7 d}$. This and $|\mathring{A}| \le |A| + \frac{1}{d} |tr A| |{\mathrm{Id}}| \le |A| + \frac{1}{d} \sqrt{d} |A| \sqrt{d}$ give $|\mathring{R}_{n+1} (t)|_{L^1} \le \frac{\delta _{n+1}}{2^7 d}$, which is the assumption (12) of the step $n+1$. Similarly, for any $t \in [0,1]$ at the *n*-th step we get, by (14)

$$\begin{aligned} \frac{3}{8} \delta _n e(t) \le e(t) - \Bigg ( \int |u_{n+1}|^2 (t) + 2 \int _0^t \int \mathcal {A} (D u_{n+1}) D u_{n+1} \Bigg ) \le \frac{5}{8} \delta _{n} e(t) \end{aligned}$$

which is the assumption (11) of the step $n+1$, since

$$\begin{aligned} \frac{3}{8} \delta _n e(t) = \frac{3}{4} \delta _{n+1} e(t), \qquad \frac{5}{8} \delta _{n} e(t) = \frac{5}{4} \delta _{n+1} e(t). \end{aligned}$$

Consequently we obtain iteratively, as $\eta _n \le 2^{-n}$,

120 $$\begin{aligned} \begin{aligned} \sup _{t \in [0,1]} |(u_{n+1} - u_n) (t)|_{L_2}&\le M_0 2^{-n/2}, \\ \sup _{t \in [0,1]} |(u_{n+1} - u_n) (t)|_{W^{1,q}}&\le 2^{-(n+1)}, \\ \sup _{t \in [0,1]} |R_{n+1} (t)|_{L_1}&\le 2^{-(n+1)} \end{aligned} \end{aligned}$$

Inequalities (121) mean that $\{u_n\}_{n=0}^\infty$ is a Cauchy sequence in $C(L^2) \cap C (W^{1,q})$. Denote its limit by $v \in C(L^2) \cap C (W^{1,q})$. Send $n \rightarrow \infty$ in the distributional formulation of (10). In particular, in order to pass to the limit in the dissipative term, take a test function $\varphi$ and use (15) for $q< \frac{2d}{d+2} <2$ and the Hölder inequality to obtain

$$\begin{aligned}&\left| \int _0^t \int \left[ \mathcal {A} (Du_n) - \mathcal {A} (Dv) \right] \nabla \varphi dxdt \right| \\&\quad \le C \left\{ \begin{aligned}&|\nabla \varphi |_{L^1_t L^q_x} \sup _{t \in [0,1]} |Du_n - Dv|_{L^q}^{q-1}(t)&\text { for } \nu _0=0, \\&|\nabla \varphi |_{L^1_t L^{q'}_x} \sup _{t \in [0,1]} |Du_n - Dv|_{L^q}(t)&\text { for } \nu _0>0. \end{aligned} \right. \end{aligned}$$

The right-hand sides tend to 0 as $n \rightarrow \infty$ thanks to (121). Consequently we see that *v* satisfies the distributional formulation of (1).

For the $2\int _0^t \int \mathcal {A} (D u_n) D u_n$ term of energy we use (16) to write

$$\begin{aligned} \int _0^t \int |\mathcal {A} (Du_n)Du_n - \mathcal {A} (Dv)Dv | \le C\int _0^t \int \big (1 + |Du_n|^{q-1} + |Dv|^{q-1} \big ) |Du_n-Dv|. \end{aligned}$$

which via Hölder inequality and (121) allows to pass with $n \rightarrow \infty$. This and $\lim _{n \rightarrow \infty } |u_{n} - v|_{C(L^2)} = 0$ provided by (121) yields (7).

Let us now focus on proving the last part of Theorem A, i.e. the non-uniqeness statement. Let us take the two energy profiles $e_1, e_2$ and the respective triples $(u^1_n, \pi ^1_n, R^1_n)$ and $(u^2_n, \pi ^2_n, R^2_n)$ of our convex integration scheme (in what follows, superscripts denote the cases of $e_1, e_2$, respectively). At each iteration step $n \rightarrow n+1$ one picks value of $\lambda ^i_n$ ($=\lambda$ of Sect. 7.3) that works for $(u^i_n, \pi ^i_n, R^i_n)$. Observe that choosing $\bar{\lambda }_n = \max {(\lambda _n^1, \lambda _n^2)}$ works simultaneously for both triples. Thus, without renaming the triples, let us make the choice $\bar{\lambda }_n$ for both $(u^i_n, \pi ^i_n, R^i_n)$, $i=1,2$. It results in using identical Mikado flows $W^k$ for both iterations.

Now we want to inductively argue that, thanks to the assumed $e_1(t) = e_2(t)$ for $t \in [0, T]$, it holds $u^1_{n} (t) = u^2_n (t)$ for every *n* and $t \in [0, T-\frac{1}{2^7 d}]$. Let us assume thence that $u^1_{n} (t)= u^2_n(t)$ and $\mathring{R}_n^1 (t)= \mathring{R}_n^2 (t)$ for times $t \in [0, T - \sum _{i=0}^n \frac{2^{-i}}{2^7 d}]$ (This holds for $n=0$, since we begin with the zero triple). Formula (47), with (44) and (45) shows that $a^i_{k,n+1} (t)$ (i.e. every $a^i_k(t)$, $k \in K$ at the step $n \rightarrow n+1$) depends on *e*(*t*), $\mathring{R}_n^{i,\epsilon } (t)$ and $u^i_{n|[0,t]}$, with $\epsilon \le \frac{2^{-(n+1)}}{2^7 d}$ being the mollification parameter, cf. (69) with the choice $\delta _{n+1} := {2^{-(n+1)}}$, and the $u^i_n$-dependence being nonlocal due to the dissipative term in (45). So by our inductive assumption we see that $a^1_{k,n+1} (t) = a^2_{k,n+1}(t)$ for times $t \in [0, T - \sum _{i=0}^{n+1} \frac{2^{-i}}{2^7 d}]$. Consequently, via the definition (49), the principal perturbations $u_{p}^i (t)$, $i=1,2$ at the step $n \rightarrow n+1$ are identical for times $t \in [0, T - \sum _{i=0}^{n+1} \frac{2^{-i}}{2^7 d}]$. Therefore $u^1_{n+1} (t)= u^2_{n+1} (t)$ and $\mathring{R}_{n+1}^1 (t)= \mathring{R}_{n+1}^2 (t)$ for $t \in [0, T - \sum _{i=0}^{n+1} \frac{2^{-i}}{2^7 d}]$, since the correctors and the new errors are defined pointwisely in time.

Under the assumption that $e_1, e_2$ are identical on [0, *T*], we produced iteratively $u^1_{n} (t), u^2_n (t)$ that agree for $t \in [0, T - \frac{1}{2^7 d}]$ thus also their limits satisfy $v^1 (t)\equiv v^2 (t)$ for $t \in [0, T - \frac{1}{2^7 d}]$.

Replacing $T - \frac{1}{2^7 d}$ with any fixed number $T_1$ strictly smaller than *T* requires only mollifying at the scales below $T-T_1$ instead of $\frac{1}{2^7 d}$.

## Sketch of the Proof of Theorem B

Let us indicate changes needed in proofs of Proposition 1 and Theorem A to reach Theorem . Now, we extend the allowed range of growths of $\mathcal {A}$ to $q \in (1, \frac{3d+2}{d+2})$ at the cost of abandoning the control over the dissipative term $2 \int _0^t \int \mathcal {A} (D v) D v$ of the energy. Recall that $r \in (\max \{1, q-1\}, \frac{2d}{d+2})$ is an additional exponent, fixed in the assumptions of Theorem B. The main observation is that

$$\begin{aligned} q-1 \le r <\frac{2d}{d+2} \end{aligned}$$

is subcritical in the sense of choices made in Sect. 7.1.

Let us first consider modifications in proof Proposition 1. Replacing in Sects. 7.1, 7.2*q* of Proposition 1 with *r* implies the following analogue of (111):

$$\begin{aligned} L(r) \le C\lambda ^{-\zeta }, \quad L(2) \le C\lambda ^{-\zeta }, \quad \lambda _2 L({r}) \le C\lambda ^{-\zeta }. \end{aligned}$$

for some positive $\zeta >0$. Consequently, (114) holds now also with *r* in place of *q*. Next, since *q* now may exceed 2, to control $|R_\mathcal {A} (t)|_1$ we use the entire (93) to write

$$\begin{aligned} |R_\mathcal {A} (t)|_1 \le \left\{ \begin{aligned}&|R_{\mathcal {A} }(t)|_{r} \le C \Big ( {\big ( \lambda _2 \mu ^{\frac{d}{2} - \frac{d}{r}} \big )^{q-1}} + (\lambda _2 L(r))^{q-1} \Big )&\text { for } \nu _0=0, \;q\le 2,\\&|R_{\mathcal {A} }(t)|_{r} \le C \big ( {\lambda _2 \mu ^{\frac{d}{2} - \frac{d}{r}}} + \lambda _2 L(r) \big )&\text { for } \nu _0>0, \;q\le 2,\\&|R_{\mathcal {A} }(t)|_{{1}} \le C \Big ( { \lambda _2 \mu ^{\frac{d}{2} - \frac{d}{q-1}}} + { \big ( \lambda _2 \mu ^{\frac{d}{2} - \frac{d}{q-1}} \big )^{q-1}} \\&\qquad \qquad \qquad \qquad + \lambda _2 L(q-1) + (\lambda _2 L(q-1))^{q-1} \Big )&\text { for } q > 2. \end{aligned} \right. \end{aligned}$$

In any of these cases, right-hand sides are controlled by powers of $\lambda _2 \mu ^{\frac{d}{2}-\frac{d}{r}}$ and $\lambda _2 L(r)$, therefore we can reach (119). Finally, since we abandon the control over the dissipative term in the energy inequality, (45) and $\delta E$ of (95) (let us rename it to $\delta \tilde{E}$) loose their dissipative terms. The consequence of the latter is that (96) simplifies to

121 $$\begin{aligned} \delta \tilde{E}(t) \le \frac{\delta }{16} e(t) + C_N \big ({\lambda ^{-1}_1} + {\mu ^{-\frac{d}{2}}} + L(2) + L(2)^2 \big ). \end{aligned}$$

Inequality (122) allows to prove (120) for $\delta \tilde{E}$ as in Sect. 7.

The above modifications allow to prove Theorem B along Sect. 8 with the difference that now $\{u_n\}_{n=0}^\infty$ forms a Cauchy sequence in $C(L^2) \cap C (W^{1,r})$ and, in the case $q >2$, we pass to the limit in the dissipative term via (15) for $q \ge 2$ and the Hölder inequality that give

$$\begin{aligned} \Bigg | \int _0^t \int [ \mathcal {A} (Du_n) - \mathcal {A} (Dv) ] \nabla \varphi \Bigg | \le C |Du_n-Dv|_{L^{q-1}} \bigg (1 + |Du_n|^{q-2}_{L^{q-1}} + |Dv|^{q-2}_{L^{q-1}} \bigg ). \end{aligned}$$

## Sketch of the Proof of Theorem C

Let us first introduce the following modification of Definition 2

### Definition 3

Fix $a \in L^2 (\mathbb {T}^d)$. A solution to *the Non-Newtonian-Reynolds Cauchy problem* is a triple $(u,\pi ,R)$ where

$$\begin{aligned} u \in L^\infty (L^2) \cap L^q (W^{1,q}), \quad \pi \in \mathcal {D}, \quad R \in L^1 \end{aligned}$$

with spatial null-mean *u*, solving the Cauchy problem

122 $$\begin{aligned} \begin{aligned} \partial _{t}u + \mathrm{div} \,(u\otimes u) - \mathrm{div} \,\mathcal {A} (Du ) +\nabla \pi&= -\mathrm{div} \,\mathring{R}, \\ \mathrm{div} \,u&= 0, \\ u(0)&= a, \end{aligned} \end{aligned}$$

in the sense of distributions, where the data are attained in the weak $L^2$-sense.

The drop of regularity between objects of Definition 3 and objects of Definition 2 stems from a different starting point for our iterations. To prove Theorems A, B, we started the iteration at the smooth triple $(u_0, \pi _0, R_0)$ = (0, 0, 0) and added smooth perturbations in each iteration. To prove Theorem C we will start iterations with $(v_a, \tilde{\pi }_a, - v_a \otimes v_a)$, where $v_a, \tilde{\pi }_a$ solves the Cauchy problem of a non-Newtonian-Stokes system:

123 $$\begin{aligned} \begin{aligned} \partial _{t}v_a - \mathrm{div} \,\mathcal {A} ( D v_a)+\nabla \tilde{\pi }_a&= 0 \\ \mathrm{div} \,v_a&= 0&\\ v_a (0)&= a \end{aligned} \end{aligned}$$

The smoothness of such $v_a, \tilde{\pi }_s$ is in general false, so even though at each step we again add smooth perturbations, the regularity at each step cannot be better than that of $v_a, \tilde{\pi }_a$ solving (124).

The starting point of our iterations is given by

### Proposition 15

(Leray–Hopf solutions for non-Newtonian Stokes). Fix $a \in L^2 (\mathbb {T}^d)$. There is

$$\begin{aligned} v_a \in L^\infty ( L^2) \cap L^q (W^{1,q}), \quad \tilde{\pi }_a \in \mathcal {D} \end{aligned}$$

solving the Cauchy problem (124) in the sense of distributions, so that $\lim _{t \rightarrow 0} |v_a (t) - a|_2 = 0$ and

$$\begin{aligned} \int _{\mathbb {T}^{d}}|v_a|^2 (t) + 2 \int _0^t \int _{\mathbb {T}^{d}}\mathcal {A} (D v_a) D v_a \le \int _{\mathbb {T}^{d}}|a|^2. \end{aligned}$$

The proof uses monotonicity of $\mathcal {A}$ and for strong attainment of the initial datum, the energy inequality.

The main ingredient of proof of Theorem C is a version of Proposition 1 tailored to deal with the Cauchy problem.

Roughly speaking, given a solution to the Non-Newtonian-Reynolds system (123), which assume the given initial datum, we construct another solution to (123) *with the same initial datum*, and with a well-controlled Reynolds stress. The price we pay to keep the initial datum intact is growth of energy. More precisely, energy of the ultimately produced solution to the Cauchy problem with datum *a* for (1) is *much above* an energy of a non-Newtonian Stokes emanating from the same *a*. Hence we cannot reach energy inequality, even for merely the kinetic energy in the range $q< \frac{2d}{d+2}$. This is why we do not distinguish the subcompact range $q< \frac{2d}{d+2}$ in Theorem C.

We are ready to state

### Proposition 16

Let $\nu _0, \nu _1 \ge 0$ and $q< \frac{3d+2}{d+2}$, $r \in (\max \{1, q-1\}, \frac{2d}{d+2})$ be fixed. Fix an arbitrary nonzero initial datum $a \in L^2({\mathbb {T}^{d}})$, $\mathrm{div} \,a = 0$. There exist a constant *M* such the following holds.

Let $(u_0, \pi _0, R_0)$ be a solution to the Non-Newtonian-Reynolds Cauchy problem with datum *a*. Let us choose any $\delta , \eta , \sigma \in (0,1]$ and $\gamma >0$. Assume that

124 $$\begin{aligned} |\mathring{R}_0 (t)|_{L^1} \le \frac{\delta }{2^7 d} \quad \text {for all } t \in [2\sigma , 1]. \end{aligned}$$

Then, there is a solution $(u_1, \pi _1, R_1)$ to Non-Newtonian-Reynolds Cauchy problem with same datum *a* such that

125 $$\begin{aligned} |(u_1 - u_0)(t)|_{L^2}&\le {\left\{ \begin{array}{ll} M \delta ^\frac{1}{2} + M \gamma ^\frac{1}{2} &{} t \in [4\sigma ,1], \\ M (\delta + \sup _{\tau \in (t-\sigma /4, t+\sigma /4)} |\mathring{R}_0(\tau )|_1 + \gamma )^\frac{1}{2} &{} t \in \left[ \sigma /2, 4 \sigma \right] , \\ 0 &{} t \in [0, \sigma /2], \end{array}\right. } \end{aligned}$$

126a $$\begin{aligned} |(u_1 - u_0)(t) |_{W^{1,r}}&\le {\left\{ \begin{array}{ll} \eta &{} \text {for all } t \in [0, 1], \\ 0 &{} t \in [0, \frac{\sigma }{2}]. \end{array}\right. } \end{aligned}$$

126b $$\begin{aligned} |R_1 (t)|_{L^1}&\le {\left\{ \begin{array}{ll} \eta , &{} t \in [\sigma , 1], \\ |R_0(t)|_{L^1} + \eta , &{} t \in [\sigma /2, \sigma ], \\ |R_0(t)|_{L^1}, &{} t \in [0, \sigma /2]. \end{array}\right. } \end{aligned}$$

and

126c $$\begin{aligned} \left| |u_1|_2^2 - |u_0|_2^2 - d \gamma \right| (t) \le \frac{\delta }{2^4} \qquad t \in [4\sigma , 1], \end{aligned}$$

### Proof

(Sketch of the proof of Proposition 16) Let us indicate the changes we need to make in the proof of Proposition 1.

The constant $\gamma$ will be used instead of the energy pump $\gamma _0 (t)$ of (45). This changes (44) and gives

$$\begin{aligned} \varrho (x,t) \le 2 \epsilon + 2 |\mathring{R}_0^\epsilon (x,t)| + \gamma \end{aligned}$$

and thus alters (73) to

127 $$\begin{aligned} \left| a_k (t) \right| _2 \le 2 ( \delta + |\mathring{R}_0(t)|_1 + \gamma )^\frac{1}{2}. \end{aligned}$$

Define $u_p$, $u_c$ by (49), (57) respectively (with the new $\varrho$). Let us introduce a smooth cutoff

$$\begin{aligned} \chi (t) {\left\{ \begin{array}{ll} =0 &{} t \le \sigma /2, \\ \in [0,1] &{} t \in (\sigma /2, \sigma ) \\ =1, &{} t \ge \sigma . \end{array}\right. } \end{aligned}$$

and define the perturbations $\tilde{u}_p$, $\tilde{u}_c$ as follows

$$\begin{aligned} \tilde{u}_p(t) := \chi (t) u_p(t), \qquad \tilde{u}_c(t) := \chi ^2(t) u_c(t). \end{aligned}$$

Due to (128), the new version of (77) reads

128 $$\begin{aligned} |u_{p}(t)|_2 \le M ( \delta + |\mathring{R}_0(t)|_1 + \gamma )^{1/2} + \lambda _1^{-\frac{1}{2}} C. \end{aligned}$$

Since (125) holds only on $[2\sigma ,1]$, and recalling the fact that $\mathring{R}_0^\epsilon$ is the mollification in space *and* time of $\mathring{R}_0$, we can follow the lines of Proposition 1 only on $[4\sigma , 1]$. This gives the first line of (126a). Concerning the case $[\sigma /2, 4\sigma ]$ of (126a) let us compute, using (128)

$$\begin{aligned} |\tilde{u}_p (t)|^2_{L^2} \le |u_p (t)|^2_{L^2} \le M ( \delta + |\mathring{R}_0(t)|_1 + \gamma ) \end{aligned}$$

Our assumption now does not control $\mathring{R}^\epsilon _0(t)$ for $t \le 2\sigma$, but we can always write, choosing $\epsilon \ll \sigma$

$$\begin{aligned} |\tilde{u}_p (t)|^2_{L^2} \le M \Big (\delta + \sup _{\tau \in (t-\sigma /4, t+\sigma /4)} |\mathring{R}_0(\tau )|_1 + \gamma \Big ), \end{aligned}$$

which, together with the smallness of $|\tilde{u}_c (t)|_{L^2}$, cf. (80), gives the case $[\sigma /2, 4\sigma ]$ of (126a). On $[0, \sigma /2]$, it holds $u_1 = u_0$, as there $\chi \equiv 0$, thence the respective part of (126a).

The estimate (126b) holds on the whole time interval, because the Mikado flows are small in $W^{1,r}$ for $r < \frac{2d}{d+2}$ by construction.

The estimate on the new Reynolds stress (126c) on $[\sigma ,1]$ is analogue to the corresponding estimate (13c) of Proposition 1, as on $[\sigma ,1]$ the time cutoff $\chi \equiv 1$. The estimate on $[0, \sigma /2]$ is trivially satisfied, as on this time interval the cutoff $\chi \equiv 0$ (here $u_0 = u_1$, so $R_0 = R_1$). On the intermediate time interval $[\sigma /2,\sigma ]$, $R_0$ is decomposed as $R_0 = R_0(1 - \chi ^2) + R_0 \chi ^2$. The term $R_0 \chi ^2$ is canceled by $\tilde{u}_p \otimes \tilde{u}_p = \chi ^2 (u_p \otimes u_p)$, as in the proof of Proposition 1, thus giving the $\eta$ in the second line of (126c), whereas the term $R_0(1 - \chi ^2)$ is responsible for $|R_0(t)|_{L^1}$ in the second line of (126c). There is, however, in the new Reynolds stress $R_1$ an additional term coming from the time derivative of the cutoff:

129 $$\begin{aligned} \mathrm{div} \,R_{cutoff}(t) := \chi '(t) u_p(t) + (\chi ^2)'(t) u_c(t). \end{aligned}$$

For the first term in (130) we just use (75) with $r=1$:

$$\begin{aligned} \begin{aligned} |\mathrm{div} \,^{-1} \big ( \chi '(t) u_p(t) \big )|_1 \le |\chi '(t) u_p(t)|_1 \le \frac{C}{\sigma } \mu ^{-d/2} \le \frac{C}{\sigma } \lambda ^{-a d/2}, \end{aligned} \end{aligned}$$

where we used the choice $\mu = \lambda ^a$, as in Sect. 7. For the second term in (130) we have, invoking (19)

$$\begin{aligned} |\mathrm{div} \,^{-1} \big ( (\chi ^2)'(t) u_c(t) \big )|_1 \le |\mathrm{div} \,^{-1} \big ( (\chi ^2)'(t) u_c(t) \big )|_2 \le |(\chi ^2)'(t) u_c(t)|_2 \le \frac{C}{\sigma } L(2) \end{aligned}$$

Since by (111) $L(2) \le C \lambda ^{-\zeta }$, both terms of (130) are estimated by negative powers of $\lambda$. Thus they can be made as small as we wish by picking $\lambda$ big enough.

Let us now justify (127) along the proof of Proposition 14. In (98) $\gamma _0$ changes to $\gamma$. Now we do not control $|\mathring{R}^\epsilon _0(t)|_1$ on the entire time interval [0, 1], only on $[4 \sigma ,1]$ via (125). On this interval $\chi =1$ and hence for any $t \in [4 \sigma ,1]$ one has the following counterpart of (102)

130 $$\begin{aligned} \left| |\tilde{u}_p|_2^2 - d \gamma \right| \le \frac{\delta }{2^5} + C \Big ( \frac{1}{\lambda _1} + \frac{\lambda _1 \mu }{\lambda _2} \Big ). \end{aligned}$$

We use now $u_1 = u_0 + \tilde{u}_p + \tilde{u}_c$ to write for any $t \in [4 \sigma ,1]$

131 $$\begin{aligned} \left| |u_1|_2^2 - |u_0|_2^2 - d \gamma \right| \le \frac{\delta }{2^5} + C \Big ( \frac{1}{\lambda _1} + \frac{\lambda _1 \mu }{\lambda _2} \Big ) +\Big | |\tilde{u}_c|_2^2 + 2 \int (u_0 \tilde{u}_c + u_0 \tilde{u}_p + \tilde{u}_p \tilde{u}_c) \Big |. \end{aligned}$$

The r.h.s. above can be made arbitrarily small in view of (131) and arguments analogous to that leading to Proposition 1, which yields (127). $\square$

Iterating Proposition 16, we can now complete the proof of Theorem C. Let us choose $\sigma _n := 2^{-n}$ along the iteration. We will choose $\delta _n = {2^{-n}}$ and $\eta _n = {2^{-(n+9)}}d^{-1}$ as in proofs of Theorems A, B. There are two main differences between the current iterations and the iterations leading to Theorems A, B. Firstly, we initiate the iterations with the triple $(v_a, \tilde{\pi }_a, - v_a \otimes v_a)$, where $v_a, \tilde{\pi }_a$ are given by Proposition 15. Secondly, we will choose the now additional free parameter $\gamma$ just to distinguish between different solutions. Namely, let us choose $\gamma _n = d^{-1} \delta _n$ *except for* $\gamma _3$, which we require it to be a large constant, say *K*.

The condition (125) for the initial triple is void (empty interval where it shall hold) and over iterations it is satisfied thanks to the first case of (126c) and our choices for $\eta _n, \delta _n, \sigma _n$. The third iteration produces $u_3$ out of $u_2$ such that

$$\begin{aligned} \left| |u_3|_2^2 - |u_2|_2^2 - d K \right| (t) \le {2^{-7}} \qquad t \in [1/2, 1]. \end{aligned}$$

At this step the energies of the iteratively produced solutions branch: choosing two *K*’s that considerably differ, we will see that the kinetic energies on $t \in [1/2, 1]$ of the finally produced solutions differ considerably.

From step $n=4$ onwards $\gamma _n = \delta _n$, thus the first line of (126a) is analogous to (13a). Iterating Proposition 16 we thus obtain convergence of the sequence $\{u_n-v_a\}_n$ to some

$$v_\infty \in C((0,1]; L^2({\mathbb {T}^{d}})) \cap C([0,1]; W^{1,r}({\mathbb {T}^{d}})).$$

Note the open side of an interval above. Taking into account the regularity class of $v_a$ and $r<q$, we thus have

$$\begin{aligned} u_n \rightarrow v_a + v_\infty :=v \qquad \text { strongly in } \quad L^\infty ((0,1); L^2({\mathbb {T}^{d}})) \cap L^r((0,1); W^{1,r}({\mathbb {T}^{d}})), \end{aligned}$$

which allows to pass to the limit in the distributional formulation of (124), since by choice $r>\max {(1, q-1)}$.

Concerning the attainment of the initial datum *a*, for any $q_0 <2$ the estimate (126b) yields $|v_\infty |_{q_0} (t) \rightarrow 0$ as $t \rightarrow 0$. Therefore $v = v_\infty + v_a$ satisfies $|v - a|_{q_0} (t) \rightarrow 0$ as $t \rightarrow 0$. From this and the fact that the $L^2$ norm of *v*(*t*) is uniformly bounded in time on [0, 1], it follows that $v(t) \rightharpoonup a$ weakly in $L^2$ as $t \rightarrow 0^+$.

Let us finally argue for multiplicity of solutions. At the step $n=3$ let us choose two different *K*, $K'$. Let us distinguish the resulting $u_n$’s and their limits *v* by, respectively, $u_n$ and $u'_n$, and *v* and $v'$. On $t \in [1/2, 1]$ (127) yields for $n \ge 4$

$$\begin{aligned} \left| |u_{n}|_2^2 - |u_{n-1}|_2^2 \right| (t) \le 2^{-(4+n)} + 2^{-n} \qquad \left| |u'_n|_2^2 - |u'_{n-1}|_2^2 \right| (t) \le 2^{-(4+n)} + 2^{-n}, \end{aligned}$$

whereas

$$\begin{aligned} \left| |u_3|_2^2 - |u_2|_2^2 - d K \right| (t) \le {2^{-7}} \qquad \left| |u'_3|_2^2 - |u'_2|_2^2 - d K' \right| (t) \le {2^{-7}} \end{aligned}$$

So, since $$u_2 = u'_2$$

$$\begin{aligned} \left| |u_{n}|_2^2 - |u_2|_2^2 - d K \right| (t) \le 1/2 \qquad \left| |u'_n|_2^2 - |u_2|_2^2 - d K' \right| (t) \le 1/2. \end{aligned}$$

The same inequalities hold for the strong limits *v*, $v'$. Therefore, for $d|K-K'|>1$ they must differ.

**Funding**   Open Access funding enabled and organized by Projekt DEAL.

## 11. Appendix

### 11.1. Proof of Lemma 1

Let us first consider a scalar function $f: \mathbb {R}\rightarrow \mathbb {R}$

$$\begin{aligned} f(t) := (\nu _0 + \nu _1 |t|)^{q-2} t. \end{aligned}$$

It is Lipschitz for $\nu _1 =0$. In the range $q \in (1,2]$ *f* is $(q-1)$-Hölder continuous for $\nu _0 = 0$, $\nu _1 >0$; and locally Lipschitz for $\nu _0 > 0$, $\nu _1 >0$. By the last statement we mean that

132 $$\begin{aligned} |f(t) - f(s)| \le C_q |t-s| (\nu _0+\nu _1 (|t|+ |s|))^{q-2}. \end{aligned}$$

It is proven by writing

133 $$\begin{aligned} \begin{aligned} |f(t) - f(s) |&\le |q-1| |t-s| \int _0^1 (\nu _0+\nu _1 |s+ x (t-s)|)^{q-2} dx \\&\le |q-1| |t-s| (\nu _0+\nu _1 (|t|+ |s|))^{q-1-\delta } \int _0^1 (\nu _0+\nu _1 |s+ x (t-s)|)^{\delta -1} dx, \end{aligned} \end{aligned}$$

with the second inequality given by splitting $q-2 = (q-1-\delta ) + (\delta -1)$ so that $q-1-\delta >0$ and thus the function $t \rightarrow t^{q-1-\delta }$ is increasing. For the integral in the second line of (134) we use that for $\gamma \in (-1, 0]$ and $|t| + |s|>0$ it holds

134 $$\begin{aligned} \int _0^1 (\nu _0+\nu _1 |s+ x (t-s)|)^{\delta -1} dx \le C_\gamma (\nu _0+\nu _1 (|t|+ |s|))^{\gamma }. \end{aligned}$$

Computation for (135) is contained in proof of Lemma 2.1 in [1]. Using (135) in (134) we obtain (133).

In the range $q \ge 2$ for *f*, the function $t \rightarrow t^{q-2}$ is increasing and integrable at 0, so (133) holds for any $\nu _0 \ge 0$, $\nu _1 \ge 0$. Altogether

135 $$\begin{aligned} |f(t) - f(s) | \le \left\{ \begin{aligned}&C_{\nu _1} |t-s|^{q-1}&\text { for } \nu _0=0, q<2 \\&C_{\nu _0} |t-s|&\text { for } \nu _0>0, q<2 \\&C_{q, \nu _0, \nu _1} |t-s| \left( 1 + |t|^{q-2} + |s|^{q-2} \right)&\text { for } q\ge 2 \end{aligned} \right. \end{aligned}$$

Replacing scalars with tensors in (136) will give (15). Details follow.

Let us consider first the (global Hölder) case $\nu _0=0, q<2$, i.e. the first line of (136). We show the respective first line of (15) by considering two cases: (i) *Q*, *P* lie along a line passing through the origin and (ii) *Q*, *P* lie on a sphere centered at the origin. The case (i) is $Q = t Q_0$, $P = s Q_0$ for some $s,t \in \mathbb {R}$ and $|Q_0| = 1$, thus (15) here follows immediately from (136). The case (ii) is $|Q| = |P| = l$ for some $l > 0$. Here

$$\begin{aligned} \mathcal {A} (Q) =Q (\nu _1 l)^{q-2}, \quad \mathcal {A} (P) = P (\nu _1 l)^{q-2}, \end{aligned}$$

so

$$\begin{aligned} \frac{|\mathcal {A} (Q) - \mathcal {A} (P)|}{|Q-P|^{q-1}} = \frac{|Q-P|}{(\nu _1 l)^{2-q} |Q-P|^{q-1}} = \Big (\frac{|Q-P|}{\nu _1 l} \Big )^{2-q} \le \Big ( \frac{2l}{\nu _1 l} \Big )^{2-q} \end{aligned}$$

and the latter is a *l*-independent constant. Both cases (i), (ii) yield for every nonzero *Q*, *P*

$$\begin{aligned} \begin{aligned} |\mathcal {A} (Q) - \mathcal {A} (P)|&\le \underbrace{\Big | \mathcal {A} (Q) - \mathcal {A} \Big ( \frac{|Q|}{|P|} P \Big ) \Big |}_{\text {arguments lie: on the sphere } \partial B_{|Q|} (0)} + \underbrace{\Big | \mathcal {A} \Big ( \frac{|Q|}{|P|} P \Big ) - \mathcal {A} (P) \Big |}_{\text {on the same line through origin}} \\&\le C_{\nu _1} \Big ( \Big | Q - \frac{|Q|}{|P|} P \Big |^{q-1} + \Big | \frac{|Q|}{|P|} P - P \Big |^{q-1} \Big ) \le C_{\nu _1} |P-Q|^{q-1}. \end{aligned} \end{aligned}$$

We have just proven the first $\nu _0=0, q<2$ case of (15).

The second (global Lipschitz) case $\nu _0>0, q<2$, i.e. the tensorial version of the second line of (136), can be proven analogously. But in fact the computation leading to (133) works well when applied immediately to tensor mappings both in the case $\nu _0>0, q<2$ and $q\ge 2$, giving the remainder of (15). Estimate (16) follows from an argument that gave the case $q \ge 2$ in (15), applied to $\tilde{f}(t) = (\nu _0 + \nu _1 |t|)^{q-2} t^2$.

### 11.2. Proof of Proposition 4 on antidivergence operators

#### (i). $\mathrm {div}^{-1}: C_0^\infty (\mathbb {T}^d; \mathbb {R}^d) \rightarrow C_0^\infty (\mathbb {T}^d; {\mathcal {S}} )$)

Let $\Delta ^{-1} v$ denote the null-mean solution *u* to $\Delta u = v$ on ${\mathbb {T}^{d}}$. Recall that $D f := \frac{\nabla f + \nabla ^T f}{2}$ (symmetric gradient), and recall $P_H f := f - \nabla \Delta ^{-1} \mathrm{div} \,f$ (Helmholtz projector). Take

$$\mathrm {div}^{-1}v := D \Delta ^{-1} v + D P_H \Delta ^{-1} v,$$

which is symmetric, because *D* is symmetric. (Compare $\mathrm {div}^{-1}$ with the inverse divergence $\frac{3}{2} D \Delta ^{-1} v + \frac{1}{2} D P_H \Delta ^{-1} v - \frac{1}{2} \mathrm{div} \,\Delta ^{-1} v {\mathrm{Id}}$ of Definition 4.2 [18], which is automatically traceless. We use the simpler choice for $\mathrm {div}^{-1}$, since trace zero is provided by a pressure shift.) Since $2 \mathrm{div} \,D = \Delta + \nabla \mathrm{div} \,$, we have for $\Delta u = v$

$$\begin{aligned} \mathrm{div} \,(D u + D P_H u)= & {} \frac{1}{2} \left( \Delta u + \nabla \mathrm{div} \,u + \Delta (u - \nabla \Delta ^{-1} \mathrm{div} \,u) + \nabla \mathrm{div} \,(u - \nabla \Delta ^{-1} \mathrm{div} \,u) \right) \\= & {} \Delta u = v. \end{aligned}$$

Estimates (19), (20) follow from arguments analogous to that of [31], proof of Lemma 2.2, so we only sketch them. The estimate (19) for $p>1$ follows from Calderón-Zygmund theory, suboptimally, because $\mathrm {div}^{-1}$ is ${-1}$-homogenous. This suboptimality yields the borderline cases. In particular $p=\infty$ holds, since $\nabla \mathrm {div}^{-1}$ is 0-homogenous thus, via Sobolev embedding and Calderón-Zygmund for $\nabla \mathrm {div}^{-1}$ one has $|\mathrm {div}^{-1}f|_\infty \le C |\nabla \mathrm {div}^{-1}f|_{d+1} \le C |f|_{d+1} \le C |f|_\infty$. The other borderline case $p=1$ follows from the fact that the operator dual to $\mathrm {div}^{-1}$ is ${-1}$-homogenous and from duality argument. The claim (20) uses (19), $-1$-homogeneity of $\mathrm {div}^{-1}$ that yields

136 $$\begin{aligned} \mathrm {div}^{-1}(u_\lambda ) = \lambda ^{-1} (\mathrm {div}^{-1}u)_\lambda , \end{aligned}$$

and that we are on a torus, so the $L^p$ norms remain unchanged under oscillations.

#### (ii). ${\mathcal {R}}_N\!: C^\infty (\mathbb {T}^d; \mathbb {R}) \times C^\infty _0(\mathbb {T}^d; \mathbb {R}^d) \rightarrow C^\infty _0(\mathbb {T}^d; {\mathcal {S}} )$)

(Compare [18], Proposition 5.2 and Corollary 5.3.) We construct the two-argument improved symmetric antidivergence iteratively upon $\mathrm {div}^{-1}$. Let us commence with

137 $$\begin{aligned} \mathcal {R}_0(f,u) := \mathrm {div}^{-1}\left( f u - \fint fu \right) . \end{aligned}$$

Our aim is an antidivergence that extracts oscillations of $u_\lambda$ out of the product $fu_\lambda$. Therefore (137) suggests to apply $\mathrm {div}^{-1}$ to $u_\lambda$, and correct the remainder. Let us thence compute

138 $$\begin{aligned} \mathrm{div} \,\left( f \mathrm {div}^{-1}u \right) = f u + \sum _{k=1}^d \partial _k f ( \mathrm{div} \,^{-1} u) e_k \end{aligned}$$

and define $\mathcal {R}_1 (f, u) := f \mathrm {div}^{-1}u - \sum _{k=1}^d {\mathcal {R}}_{0} \left( \partial _k f, (\mathrm {div}^{-1}u) e_k \right) .$ The choice (138) of $\mathcal {R}_0$ and (139) yield

139 $$\begin{aligned} \mathrm{div} \,\mathcal {R}_1 (f, u) = f u - \fint fu. \end{aligned}$$

Define inductively $\mathcal {R}_N(f, u) := f \mathrm {div}^{-1}u - \sum _{k=1}^d \mathcal R_{N-1} \left( \partial _k f, (\mathrm {div}^{-1}u) e_k \right) ,$ per analogiam with the construction $\mathcal {R}_0 \rightarrow \mathcal {R}_1$. Using induction over *N*, one proves now that ${\mathcal {R}}_N$ is bilinear, symmetric, $\mathrm{div} \,\mathcal {R}_N (f, u) = f u - \fint _{\mathbb {T}^{d}}fu$, satisfies Leibniz rule:

$$\begin{aligned} \partial _k \mathcal {R}_N (f,u) = \mathcal {R}_N(f, \partial _k u) + \mathcal {R}_N( \partial _k f, u) \end{aligned}$$

and estimates (21), similarly to proof of Lemma 3.5 of [30]. For instance, to show $\mathrm{div} \,\mathcal {R}_N (f, u) = fu - \fint _{\mathbb {T}^{d}}fu$ we have (140) as the initial step. Assuming $\mathrm{div} \,\mathcal {R}_{N-1} (f, u) = fu - \fint _{\mathbb {T}^{d}}fu$, one has

$$\begin{aligned} \mathrm{div} \,\mathcal {R}_N (f, u)= & {} \mathrm{div} \,( f \mathrm {div}^{-1}u) - \sum _{k=1}^d \Big ( \partial _k f (\mathrm {div}^{-1}u) e_k - \fint \partial _k f \left( (\mathrm {div}^{-1}u) e_k \right) \Big )\\= & {} f u - \fint fu \end{aligned}$$

with the second equality due to (139).

The estimate (21) holds for $N=1$ since (19) and Jensen imply $| \mathcal {R}_0 (f, u) |_p \le C |f|_r |u|_s$ and thus

$$\begin{aligned} \begin{aligned} | \mathcal {R}_1 (f, u_\lambda ) |_{p}&\le C |f|_r |\mathrm {div}^{-1}u_\lambda |_s + \sum _{k=1}^d |{\mathcal {R}}_{0} \left( \partial _k f, (\mathrm {div}^{-1}u_\lambda ) e_k \right) |_p \\&\le C|f|_r |\mathrm {div}^{-1}u_\lambda |_s + C |\nabla f|_r | (\mathrm {div}^{-1}u_\lambda ) |_s \le C |u|_s \Big (\frac{1}{\lambda } |f|_r + \frac{1}{\lambda } |\nabla f|_r \Big ) , \end{aligned} \end{aligned}$$

with the last inequality due to (20). For the inductive step $N-1 \rightarrow N$, let us compute, using (137)

$$\begin{aligned} \begin{aligned}&|\mathcal {R}_N(f, u_\lambda )|_{p} \le C |f|_r |\mathrm {div}^{-1}u_\lambda |_s +\frac{1}{\lambda }\sum _{k=1}^d | \mathcal {R}_{N-1} \left( \partial _k f, ((\mathrm {div}^{-1}u) (\lambda \cdot )) e_k \right) |_p \\&\le C |u|_s \frac{1}{\lambda } |f|_r + C_{d,p,s,r,N-1} \sum _{k=1}^d |\mathrm {div}^{-1}u|_s \frac{1}{\lambda } \Big ( \frac{1}{\lambda } |\partial _k f|_r + \frac{1}{\lambda ^{N-1}} |\nabla ^{N-1} \partial _k f|_r \Big ), \end{aligned} \end{aligned}$$

with the second inequality valid via the inductive assumption, i.e. (21) for $N-1$. The estimate (19) and interpolation yield the inductive thesis.

#### (iii). $\tilde{\mathcal {R}}_N: C^\infty (\mathbb {T}^d; \mathbb {R}^d) \times C^\infty _0(\mathbb {T}^d; \mathbb {R}^{d \times d}) \rightarrow C^\infty _0(\mathbb {T}^d; {\mathcal {S}} )$)

Take

$$\begin{aligned} \tilde{\mathcal {R}}_N ( v, T) := \sum _{k=1}^d {\mathcal {R}}_N (v_k, T e_k ). \end{aligned}$$

Then $\mathrm{div} \,\tilde{\mathcal {R}}_N (v, T) = \sum _{k=1}^d v_k T e_k - \fint _{\mathbb {T}^{d}}v_k T e_k = Tv - \fint _{\mathbb {T}^{d}}Tv$ and since it is a linear combination of ${\mathcal {R}}_N$’s, it retains all its properties.

#### (iv). ${\mathcal {R}}^2_N: C^\infty (\mathbb {T}^d; \mathbb {R}) \times C^\infty _0(\mathbb {T}^d; \mathbb {R}) \rightarrow C^\infty _0(\mathbb {T}^d; {\mathcal {S}} )$)

We redo the reasoning of (i)–(iii), starting from the following ‘standard double antidivergence’ $\mathrm {div}^{-2}: C_0^\infty (\mathbb {T}^d; \mathbb {R}) \rightarrow C_0^\infty (\mathbb {T}^d; {\mathcal {S}} )$

$$\begin{aligned} \mathrm {div}^{-2}v := \nabla ^2 \Delta ^{-2} v, \end{aligned}$$

where $\nabla ^2$ is the (symmetric) tensor of second derivatives, and thus $\mathrm{div} \,\mathrm{div} \,\mathrm{div} \,^{-2} f = f.$ Analogously as for $\mathrm {div}^{-1}$ we have $| \mathrm {div}^{-2}v|_{W^{1,p}} \le |v|_p C_p.$ Since it is $-2$-homogenous, it holds $\mathrm {div}^{-2}(v_\lambda ) = \lambda ^{-2} (\mathrm {div}^{-2}v)_\lambda ,$ thus

140 $$\begin{aligned} | \nabla ^i \mathrm {div}^{-2}(v_\lambda )|_{L^{p}} \le \lambda ^{i-2} |\nabla ^i v|_{L^{p}} C_{k,p}. \end{aligned}$$

Upon $\mathrm {div}^{-2}$ we build now ${\mathcal {R}}^2_N$, starting with

141 $$\begin{aligned} \mathcal {R}^2_0(a,b) := \mathrm {div}^{-2}\Big (ab - \fint ab \Big ) \end{aligned}$$

and the computation

142 $$\begin{aligned} \mathrm{div} \,\mathrm{div} \,\left( a \,\mathrm {div}^{-2}b\right) = a b + \sum _{k,l=1}^d 2 \partial _{l} (\mathrm {div}^{-2}b)^{kl} \partial _k a +(\mathrm {div}^{-2}b)^{kl} \partial ^2_{kl} a, \end{aligned}$$

where we used symmetry of $\mathrm {div}^{-2}$. Hence let us define by recursion

$$\begin{aligned} \mathcal {R}^2_N (a, b) := a \,\mathrm {div}^{-2}b - \sum _{k,l=1}^d \big [ 2 \mathcal {R}^2_{N-1} \left( \partial _k a, \partial _{l} (\mathrm {div}^{-2}b)^{kl} \right) + \mathcal {R}^2_{N-1} \left( \partial ^2_{kl} a, (\mathrm {div}^{-2}b)^{kl} \right) \big ]. \end{aligned}$$

Let us inductively prove that

143 $$\begin{aligned} \mathrm{div} \,\mathrm{div} \,\mathcal {R}^2_N (a, b) = ab - \fint ab. \end{aligned}$$

The initial statement for $N=0$ is (142). Assuming (144) for $N-1$, we use (143) to compute

$$\begin{aligned} \mathrm{div} \,\mathrm{div} \,\mathcal {R}^2_N (a, b) = a b + \fint \sum _{k,l=1}^d \left( 2\partial _k a \, \partial _l (\mathrm {div}^{-2}b)^{kl} + \partial ^2_{kl} a (\mathrm {div}^{-2}b)^{kl} \right) . \end{aligned}$$

We see again via (143) that the mean value above equals $- \fint ab$.

Via induction over *N*, one proves that ${\mathcal {R}}^2_N$ is bilinear, symmetric, and satisfies Leibniz rule. Concerning the estimate (23), let us first prove it for $j=0$. The proof is by induction on *N*. For $N=1$, the estimate is true, since $| \mathcal {R}^2_0 (a, b) |_p \le C |a|_r |b|_s$ and thus

$$\begin{aligned} \begin{aligned} | \mathcal {R}^2_1 (a, b_\lambda ) |_{p}&\le C|a|_r |\mathrm{div} \,^{-2} b_\lambda |_s + C |\nabla a|_r | (\nabla \mathrm{div} \,^{-2} b_\lambda ) |_s + C |\nabla ^2 a|_r | \mathrm{div} \,^{-2} b_\lambda |_s \\&\le C |b|_s \Big ( \frac{1}{\lambda ^2} |a|_r + \frac{1}{\lambda } |\nabla a|_r + \frac{1}{\lambda ^2} |\nabla ^2 a|_r \Big ) \end{aligned} \end{aligned}$$

with the last inequality due to (141). For the inductive step $N-1 \rightarrow N$, let us compute using $-2$-homogeneity of $\mathrm {div}^{-2}$, $-1$-homogeneity of its derivatives, bililnearity of $\mathcal {R}^2$

$$\begin{aligned}&|\mathcal {R}^2_N(a, b_\lambda )|_{p} \le C |b|_s \frac{1}{\lambda ^2} |a|_r \\&\quad +\sum _{k,l=1}^d 2 \frac{1}{\lambda }| \mathcal {R}^2_{N-1} \left( \partial _k a, \partial _l (\mathrm {div}^{-2}b)^{kl}_\lambda \right) |_p + \frac{1}{\lambda ^2} | \mathcal {R}^2_{N-1} \left( \partial ^2_{kl} a, (\mathrm {div}^{-2}b )^{kl}_\lambda \right) |_p, \end{aligned}$$

using to the r.h.s. above the $j=0$ inductive assumption (23) for $N-1$ and interpolation yields (23) for $j=0$. Finally, estimate (23) for any $j \in \mathbb {N}$ is proven by induction on *j*. We already know that (23) holds for $j=0$. Assuming it is valid for *j*, one has by Leibniz rule and linearity

$$\begin{aligned} \nabla ^{j+1} {\mathcal {R}}^2_N (a, b_\lambda ) = \nabla ^{j} {\mathcal {R}}^2_N (\nabla a, b_\lambda ) +\lambda \nabla ^{j} {\mathcal {R}}^2_N (a, (\nabla b)_\lambda ), \end{aligned}$$

which via the inductive assumption yields (23) for $j+1$.

### 11.3. Proof of Lemma 3

The proof of part (i) is by induction on |*K*|. For $|K| = 1$ the proof is trivial. Let us now assume $|K| \ge 2$. Let us write $K = K' \cup \{k\}$, where $|K'| = |K| -1$. By inductive assumption, $\{\zeta _{k'}\}_{k' \in K'}$ and $\rho '>0$ are already defined, so that the periodization of the cylinders with radius $\rho '$ and axis $\{\zeta _{k'}+sk'\}_{s \in \mathbb {R}}$, for $k' \in K'$, are pairwise disjoints. It is then enough to find $\zeta _k \in {\mathbb {R}}^d$ and $\rho \in (0, \rho ')$ such that (27) holds for all $k' \in K'$. Notice that (27) is equivalent to

$$\begin{aligned} B_\rho (\zeta _k) \cap \Big ( B_\rho (0) + \big \{ \zeta _{k'} + sk + sk' \}_{s,s' \in \mathbb {R}} + {\mathbb {Z}^{d}}\Big ) = \emptyset . \end{aligned}$$

Since $d \ge 3$, the countable union of planes $\{ \zeta _{k'} + sk + s'k'\}_{s,s' \in \mathbb {R}} + {\mathbb {Z}^{d}}$ has zero measure and, since $k,k' \in {\mathbb {Z}^{d}}$, it is closed in ${\mathbb {R}}^d$. Therefore, for any $\rho \in (0, \rho ')$, also

$$\begin{aligned} \bigcup _{k' \in K'} \overline{B_\rho (0)} + \big \{ \zeta _{k'} + sk + s'k'\}_{s,s' \in \mathbb {R}} + {\mathbb {Z}^{d}}\end{aligned}$$

is closed in ${\mathbb {R}}^d$ and, if $\rho$ small enough, it is strictly contained in ${\mathbb {R}}^d$. We can thus find $\zeta _k$ and $\rho \in (0, \rho ')$ such that

$$\begin{aligned} B_\rho (\zeta _k) \subseteq {\mathbb {R}}^d\setminus \Big (\bigcup _{k' \in K'} \overline{B_\rho (0)} + \big \{ \zeta _{k'} + sk + s'k' \, : \, s,s' \in \mathbb {R}\big \} + {\mathbb {Z}^{d}}\Big ), \end{aligned}$$

with the superset being open, thus concluding the proof of part (i).

For part (ii), consider the set of directions in $\mathbb {Z}^{3}$ given by

$$\begin{aligned} \tilde{K} := \{(k,1) \, : \, k \in K \} \subseteq \mathbb {Z}^{3}. \end{aligned}$$

By part (i), we can find $\{\tilde{\zeta }_k\}_{k \in K} \subseteq \mathbb {R}^3$ and $\rho >0$ such that (in $\mathbb {R}^3$),

144 $$\begin{aligned} \Big ( B_\rho (\tilde{\zeta }_k) + s \underbrace{(k, 1)}_{\in \tilde{K}} + \mathbb {Z}^3 \Big ) \cap \Big ( B_\rho (\tilde{\zeta }_{k'}) + s' \underbrace{(k', 1)}_{\in \tilde{K}} + \mathbb {Z}^3 \Big ) = \emptyset \end{aligned}$$

for all $k,k' \in K$ and $s \in \mathbb {R}$ (the balls above are in $\mathbb {R}^3$). Observe that, for every $(k,1) \in \tilde{K}$, the axis of the cylinder $B_\rho (\tilde{\zeta }_k) + s (k, 1) + \mathbb {Z}^3 \subseteq \mathbb {R}^3$ is never lying on the horizontal plane $x_1, x_2$. Therefore we can assume w.l.o.g. that each $\tilde{\zeta }_k$ has the form

$$\begin{aligned} \tilde{\zeta }_k = (\zeta _k, 0). \end{aligned}$$

Let us now show (28), with such choice of $\{\zeta _k\}_{k \in K}$. Assume by contradiction that there are two distinct directions $k,k' \in K$ and $s \in \mathbb {R}$ such that

$$\begin{aligned} \big ( B_\rho (\zeta _k) + s k + \mathbb {Z}^2 \Big ) \cap \big ( B_\rho (\zeta _{k'}) + sk' + \mathbb {Z}^2 \Big ) \ne \emptyset . \end{aligned}$$

Then there are $x \in B_\rho (\zeta _k) \subseteq \mathbb {R}^2$, $y \in B_\rho (\zeta _{k'}) \subseteq \mathbb {R}^2$, $l,l' \in \mathbb {Z}^2$, such that

$$\begin{aligned} x + sk + l = y + sk' + l' \in \mathbb {R}^2. \end{aligned}$$

This implies that

$$\begin{aligned} \underbrace{(x,0)}_{\in B_\rho (\tilde{\zeta }_k)} + s(k,1) + \underbrace{(l,0)}_{\in \mathbb {Z}^3} = \underbrace{(y,0)}_{\in B_\rho (\tilde{\zeta }_{k'})} + s(k',1) +\underbrace{(l',0)}_{\in \mathbb {Z}^3} \in \mathbb {R}^3, \end{aligned}$$

contradicting (145).

## References

[1] Acerbi, E., Fusco, N.: Regularity for minimizers of non-quadratic functionals: The case $$1<p<2$$. J. Math. Anal. Appl. **140**(1), 115–135 (1989)

[2] Beekie, R., Buckmaster, T., Vicol, V.: Weak solutions of ideal mhd which do not conserve magnetic helicity. *Ann. PDE*, 1(1), (2020)

[3] Bird RB, Armstrong RC, Hassager O (1987). Dynamics of Polymer Liquids.

[4] Blechta J, Malek J, Rajagopal KR (2020). On the classification of incompressible fluids and a mathematical analysis of the equations that govern their motion. SIAM J. Math. Anal..

[5] Brué E, Colombo M, De Lellis C (2021). Positive solutions of transport equations and classical nonuniqueness of characteristic curves. Arch. Rational Mech. Anal..

[6] Buckmaster, T., Colombo, M., Vicol, V.: Wild solutions of the navier-stokes equations whose singular sets in time have hausdorff dimension strictly less than $$1$$. arXiv:1809.00600

[7] Buckmaster, T., De Lellis, C., Székelyhidi, L., Vicol, V.: Onsager’s conjecture for admissible weak solutions. Commun. Pure Appl. Math. **72**(2), 229–274 (2018)

[8] Buckmaster T, Shkoller S, Vicol V (2019). Nonuniqueness of weak solutions to the sqg equation. Commun. Pure Appl. Math..

[9] Buckmaster T, Vicol V (2019). Convex integration and phenomenologies in turbulence. EMS Surv. Math. Sci..

[10] Buckmaster T, Vicol V (2019). Nonuniqueness of weak solutions to the Navier–Stokes equation. Ann. Math..

[11] Bulíček, M., Kaplický, P., Pražák, v: Uniqueness and regularity of flows of non-newtonian fluids with critical power-law growth. Math. Models Methods Appl. Sci., **29**(06):1207–1225, (jun 2019)

[12] Cheskidov, A., Dai, M.: Kolmogorov’s dissipation number and the number of degrees of freedom for the 3d navier-stokes equations. Proc. Roy. Soc. Edinburgh Sect. A **149**(2), 429–446 (2019)

[13] Cheskidov, A., Luo, X.: Nonuniqueness of weak solutions for the transport equation at critical space regularity. arXiv:2004.09538

[14] Cheskidov A, Luo X (2021). Anomalous dissipation, anomalous work, and energy balance for smooth solutions of the Navier–Stokes equations. SIAM J. Math. Anal..

[15] Cheskidov A, Shvydkoy R (2014). Euler equations and turbulence: analytical approach to intermittency. SIAM J. Math. Anal..

[16] Colombo M, Lellis CD, Rosa LD (2018). Ill-posedness of leray solutions for the hypodissipative Navier–Stokes equations. Commun. Math. Phys..

[17] Daneri S, Székelyhidi L (2017). Non-uniqueness and h-principle for hölder-continuous weak solutions of the Euler equations. Arch. Ration. Mech. Anal..

[18] De Lellis C, Székelyhidi L (2013). Dissipative continuous Euler flows. Invent. math..

[19] de Waele, A.: Viscometry and plastometry. Journal of the Oil & Colour Chemists Association (1923)

[20] Diening L, Ruzicka M, Wolf J (2010). Existence of weak solutions for unsteady motions of generalized newtonian fluids. Ann. Sc. Norm. Super. Pisa Cl. Sci..

[21] Frehse J, Malek J, Steinhauer M, Jager W, Necas J, John O, Najzar K, Stara J (2000). On existence result for fluids with shear de-pendent viscosity - unsteady flows. Partial Differential Equations.

[22] Gurtin M, Fried E, Anand L (2010). The Mechanics and Thermodynamics of Continua.

[23] Isett, P.: A proof of onsager’s conjecture. Ann. Math. **188**(3), 871 (2018)

[24] Ladyzhenskaya, O.A.: On some problems from the theory of continuous media (russian). ICM Proceedings, Moscow (1966)

[25] Lellis CD, Székelyhidi L (2009). The Euler equations as a differential inclusion. Ann. Math..

[26] Luo, T., Titi, E. S.: Non-uniqueness of weak solutions to hyperviscous Navier–Stokes equations - on sharpness of J.-L. Lions exponent. Calc. Var. PDE, 59(92), (2020)

[27] Luo X (2019). Stationary solutions and nonuniqueness of weak solutions for the Navier–Stokes equations in high dimensions. Arch. Rational Mech. Anal..

[28] Malek J, Necas J, Rokyta M, Ruzicka M (1996). Weak and Measure-Valued Solutions to Evolutionary PDEs.

[29] Malek J, Necas J, Ruzicka M (1993). On the non-newtonian incompressible fluids. Math. Models Methods Appl. Sci..

[30] Modena, S., Sattig, G.: Convex integration solutions to the transport equation with full dimensional concentration. Annales de l’Institut Henri Poincaré C, Analyse non linéaire (2020)

[31] Modena S, Székelyhidi L (2018). Non-uniqueness for the transport equation with sobolev vector fields. Ann PDE.

[32] Modena S, Székelyhidi L (2019). Non-renormalized solutions to the continuity equation. Calc. Var. PDE.

[33] Norton F (1929). The Creep os Steel at High Temperatures.

[34] Ostwald, W.: Uber die rechnerische darstellung des strukturgebietes der viskositat. Kolloid-Zeitschrift (1929)

[35] Rosa LD (2019). Infinitely many Leray–Hopf solutions for the fractional Navier–Stokes equations. Commun. Partial Differ. Equ..

[36] Rudolph, N., Osswald, T.A.: Polymer Rheology. Hanser Fachbuchverlag (2014)

[37] Saramito P (2016). Complex Fluids.

[38] Schowalter W (1978). Mechanics of Non-Newtonian Fluids.

[39] Tanner (2000). Engineering Rheology.

[40] Wiedemann, E.: Existence of weak solutions for the incompressible euler equations. Annales de l’Institut Henri Poincaré C, Analyse non linéaire **28**(5), 727–730 (2011)

